# Neural imbalance between feedback sensitivity and motor inhibition in compulsivity and negative urgency

**DOI:** 10.1038/s41398-026-04098-z

**Published:** 2026-05-15

**Authors:** Raoul Wüllhorst, Rebecca Overmeyer, Kerstin Dück, Verena Wüllhorst, Tanja Endrass

**Affiliations:** Institute for Clinical Psychology and Psychotherapy, Faculty of Psychology, Technische Universität Dresden, Dresden, Germany

**Keywords:** Human behaviour, Diagnostic markers, Addiction

## Abstract

Compulsivity and emotional impulsivity (negative urgency) are considered transdiagnostic risk factors for compulsive-impulsive psychopathology that is linked to a neural imbalance between executive and motivational-emotional systems. However, existing evidence does not derive from within-subjects designs, leaving it unclear whether imbalance occurs with high expressions in compulsivity and negative urgency. To address this gap, we conducted a preregistered analysis to examine how feedback sensitivity and motor inhibition interact as a function of compulsivity and negative urgency.

Participants (*n* = 205; collected 2018–2019) expressing a wide range in compulsivity and impulsivity performed two motor inhibition and two feedback tasks during electroencephalography. We examined how the relationship between neural correlates of motor inhibition (nogo/stop>go) and feedback sensitivity (loss>gain) was moderated by compulsivity and negative urgency. Compulsivity emerged as the most robust moderator. Across multiple tasks, feedback sensitivity was associated with motor inhibitory activity in participants with mild, but not severe, compulsivity. A similar effect was observed in low versus high negative urgency. The moderation by compulsivity remained when controlling for negative urgency, and vice versa. These results suggest a neural imbalance between systems tied to motor inhibition and feedback sensitivity in transdiagnostic risk. This imbalance manifests such that strong motivational-emotional sensitivity is insufficiently compensated by motor inhibitory resources. It could drive repetitive or rash behaviors in response to distress in compulsivity and negative urgency, respectively. Whereas shared mechanisms may underlie compulsivity and negative urgency, the current interactions appear to also reflect processes unique to each construct.

## Introduction

Impulsivity is a multidimensional construct [1] encompassing disinhibited behavior with potentially undesirable consequences [2]. Compulsivity describes repetitive behavior driven by urges despite negative consequences [3]. Historically viewed as opposing poles of a continuum [4, 5], impulsivity and compulsivity are now believed to reflect distinct but overlapping dimensions [6–9]. The relevance of compulsivity and impulsivity in both addiction and obsessive-compulsive disorder (OCD) [10–12] are well-documented, suggesting that impulsivity and compulsivity affect psychopathology across diagnoses. Importantly, a striking commonality between impulsivity and compulsivity is dysregulation of negative affect [13]. In compulsivity, distress drives uncontrollable urges and repetitive behavior [14]. In impulsivity, negative urgency—a facet from the prominent UPPS model [15] describing rash acts when experiencing negative emotions—captures the effect of negative affect on executing rash behaviors [16]. Emphasizing the transdiagnostic relevance of compulsivity and negative urgency, negative urgency may be a factor for substance-related problems [17–19], and is elevated in OCD [12]. Additionally, negative urgency was correlated with compulsivity and obsessive thought [20–24].

From a dual-systems perspective, impulsivity can be construed as insufficient regulation of a hyperactive ventral striatum by the prefrontal cortex [1]. Similarly, compulsivity is thought to involve altered orbitofrontal influence on the dorsal striatum [25]. Pre- and orbitofrontal cortices belong to a frontoparietal control network subserving executive functions [26], processes involved in controlling cognition, behavior, or emotions [27]. Three supposed core executive functions are updating, flexibility, and inhibition [28, 29], the latter encompassing motor inhibition, i.e., withholding or cancelling a prepotent response [30]. Motor inhibition is thought to rely on the inferior frontal cortex, insula, and medial regions spanning the pre-supplementary motor area (pre-SMA) and dorsal midcingulate cortex together with the striatum and subthalamic nucleus of the basal ganglia [31–33]. Considering dual-systems approaches to impulsive-compulsive psychopathology more broadly, meta-analyses suggest a neurobiological imbalance between hyperactive salience signals and hypoactive prefrontal control in addictions [34, 35], along with impaired performance in motor inhibition [36, 37]. Similarly, meta-analyses show hypoactive prefrontal cortex and hyperactive salience signals in OCD, particularly during emotional processing [38, 39], as well as impairments in motor inhibition [40, 41]. Negative urgency may emulate these patterns of imbalance [42].

However, it remains a major shortcoming that imbalance models of these disorders primarily rest on converging patterns of brain activity obtained from isolated studies on particular functional domains or networks [43]. More specific tests of imbalance as the within-subjects interrelationship between different domains, have linked neural measures and self-report in substance use. Reduced neural activity tied to motor inhibition was associated with greater subjective drug reward ; [44, 45, see 46 for a review] and, together with high approach motivation, predicted earlier onset of substance use [47], a well-established risk factor for substance use disorders [48]. Substance use problems were related to low neural reward sensitivity in males with high, but not low, trait disinhibition [49]. Lower motor inhibition-related relative to reward- and emotion-related brain activity during cue exposure was tied to drinking motives [50].

Targeting neural imbalance directly, brain-brain relationships were investigated in binge-drinking [51] and binge-watching [52], both of which are linked to negative urgency [53, 54]. Weafer et al. [51] used fMRI to assess striatal feedback sensitivity with a gain > loss contrast (‘reward sensitivity’) and our group [52] used EEG to measure feedback sensitivity with a loss > gain contrast (for the purposes of this report referred to as ‘loss sensitivity’, defined as an increased electrocortical response to monetary losses compared to gains). Compatible with imbalance models, lower motor inhibitory activity (successful stop/nogo > go contrasts) was consistently related to higher reward sensitivity in binge drinkers [51] and lower loss sensitivity in high binge-watchers [52]. Using EEG, we recorded the P3b during feedback, a positive parietal deflection ~300 ms after feedback onset reflecting feedback salience and expectancy violations [55, 56], and the P3 during motor inhibition, a central positive deflection ~300 ms after nogo or stop signals reflecting the implementation of motor inhibition [57, 58]. Lower P3b for losses vs. gains was related to a lower P3 for successful motor inhibition vs. go responses in both a stop signal and a go/nogo task. This association was most prominent in those with problematic loss of control over watching, hinting at the significance of this interaction. In line with the neural correlates of motor inhibition, the P3 is tied to medial and pre-central generators, the insula, and basal ganglia in motor inhibition tasks [31]. However, the neural sources of the P3 during feedback are less clearly defined [59].

As such, in addition to meta-analytic evidence for imbalance from isolated domains, there is accumulating evidence that potentially problematic behaviors can be characterized by altered brain-brain relationships between outcome and inhibitory processing. However, to fully assess the relevance of this imbalance in psychopathology risk, it is crucial to systematically examine imbalance in transdiagnostic risk factors, a key missing link in current research. Here, we focused on compulsivity and negative urgency as transdiagnostically relevant traits associated with maladaptive behavior potentially due to an imbalance between emotional and control-related functioning.

In sum, the present work aimed to a) examine imbalance between motor inhibition and feedback processing as within-subjects associations, b) transcend a singular focus on excessive and addictive behaviors by broadening the perspective to incorporate compulsivity and negative urgency as well-established risk factors, as well as c) quantify feedback sensitivity by contrasting losses vs. gains during both a stimulus discrimination and a complex learning task. Following pre-registered hypotheses and analyses (https://osf.io/qb4jp/), we investigated whether compulsivity and negative urgency modulated the association between neural feedback and inhibitory processing. A large sample of participants performed two tasks measuring feedback processing (incentivized flanker task and two-step learning task) and two tasks assessing motor inhibition (go/nogo task and stop signal task), while EEG was recorded. We expected that feedback-related brain activity interacted with compulsivity and negative urgency in predicting brain activity related to motor inhibition. Specifically, based on our previous results in binge-watchers [52], we tested the following hypotheses: (A) The relationship between brain activity associated with feedback processing and motor inhibition is stronger in individuals scoring high on compulsivity and negative urgency than in those scoring low on these measures. (B) This brain-brain relationship should be such that higher activity for loss vs. gain is linked to higher activity for go vs. nogo and go vs. successful stop trials. Lastly, based on our prior findings, we focused on the relationships between feedback-related and motor inhibition-related P3, presenting other components in the supplementary material.

## Methods

### Sample

We recruited 252 participants with expressions of impulsivity and compulsivity ranging from the lowest to the highest ends of these dimensions, via an online survey including the Barratt Impulsiveness Scale (BIS-11, [60]) and Obsessive-Compulsive Inventory-Revised (OCI-R, [61]). Our final sample of *n* = 205 (*n* = 107; 52% male; see Supplementary Sample Information for details on sample selection) was aged *M±SD* = 25±5 years (range: 18–44); *n* = 24 (11.7%) reported a life-time psychological disorder (see Supplementary Sample Information). We obtained informed consent and compensated with 80–100 EUR prior to and after participation, respectively. This study was conducted in accordance with the Declaration of Helsinki and approved by the local ethics committee (EK 372092017).

### Tasks and procedure

**Fig. 1:**
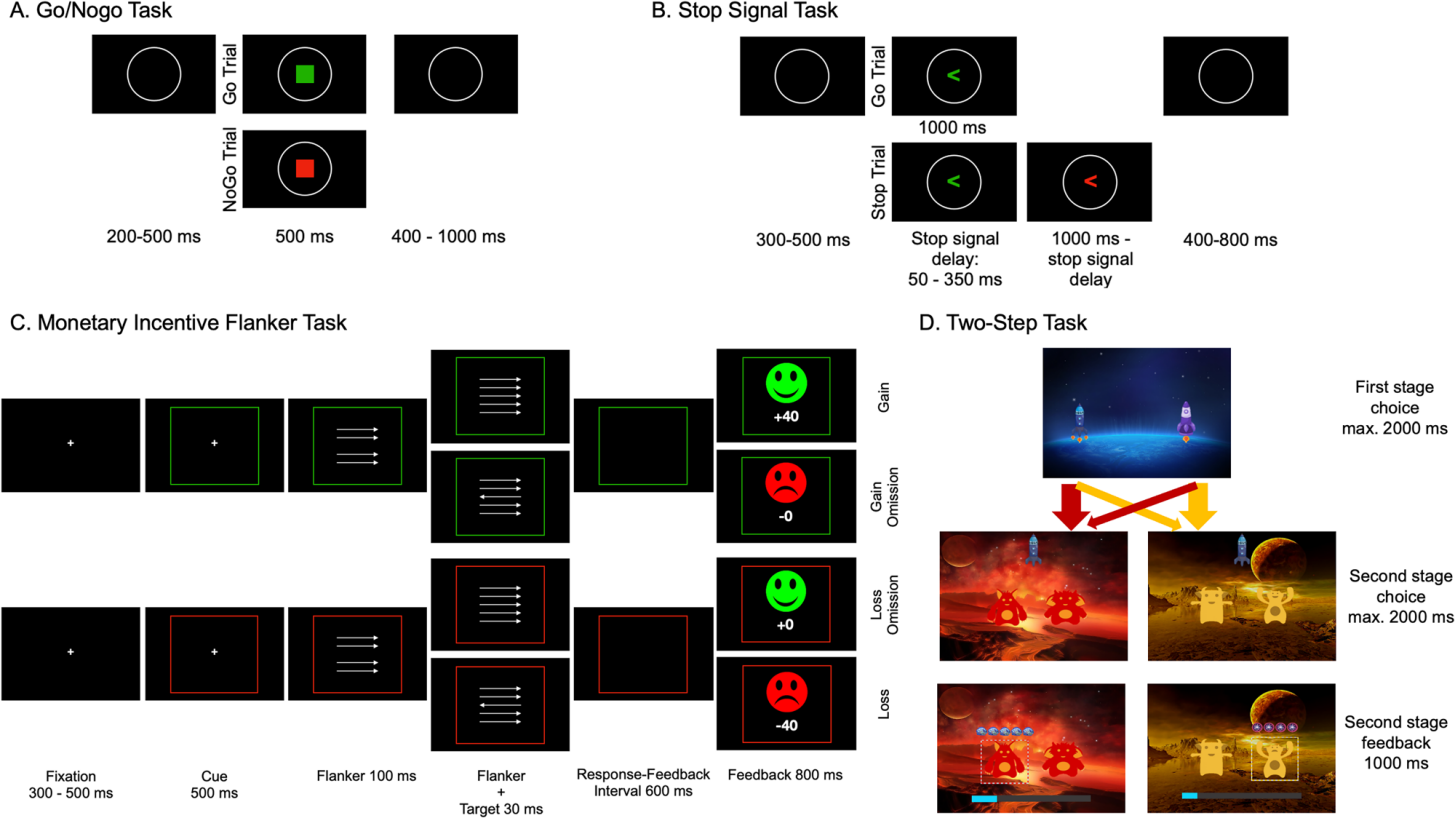
Trial schemes of the four tasks. **A** In the go/nogo task, a green square (go signal, 75% of trials) indicated to respond and a red square (nogo signal, 25%) indicated to withhold a response. **B** In the stop signal task, a green right- or left-pointing arrow (go signal) asked to indicate its direction. On stop trials (25%), the arrow turned red after the stop signal delay, asking to cancel the already initiated response. All participants completed two valid blocks (stopping accuracies between 40 and 60%). **C** In the monetary incentive flanker task, a green or red frame indicated a potential gain or loss avoidance context. Participants indicated the direction of the target arrow as quickly and accurately as possible. Performance feedback in the potential gain context rewarded fast and correct responses; errors and slow responses resulted in gain omission. In the loss avoidance context, errors and slow responses were punished, fast and correct responses resulted in loss omission. **D** In the two-step task, participants chose between two first-stage stimuli (spaceships), transitioning to one of two second-stage stimuli (planets); Each first-stage stimulus transitioned with 80% probability (common transition) to one of the two second-stage stimuli and with 20% probability (rare) to the other. In the second stage, participants encountered a pair of stimuli (aliens) specific to each planet and chose one, which added or subtracted points from their total, adhering to a random walk with reflective bounds at +5 (space treasure) and -4 (anti-matter) points. Participants were asked to respond as quickly and accurately as possible in **A**-**C**.

The overall project involved ecological momentary assessment between two laboratory sessions. Relevant to the current report, the UPPS scale was obtained during the first session and the OCI-R was assessed during the second session, when participants also performed four tasks (see Fig. 1 and Supplementary Task Description) with EEG in balanced order. We used the go/nogo task (GNGT) to assess withholding a motor response before initiation, eliciting the nogo P3. Briefly, during the GNGT (detailed in [62]), participants were asked to withhold a predominant response on 25% of trials. The stop signal task (SST) served to measure stopping an already initiated response, eliciting the stop signal P3. During the SST (detailed in [63]), participants were asked to cancel a speeded stimulus discrimination response if a stop signal occurred on 25% of trials with an adaptive delay to ensure ~50% successful stops (see Supplementary Task Description). Withholding and stopping responses show conceptual and electrophysiological overlap but are biologically and cognitively distinct [64–66], justifying the examination of both. We used neural P3 responses to performance (speed and accuracy) feedback in a monetary incentive flanker task (MIFLAT) and choice feedback in a two-step task to quantify loss sensitivity (loss vs. gain feedback). The MIFLAT (detailed in [52]) featured a potential gain context—fast and correct responses were rewarded whereas errors and slow responses resulted in reward omission—and a loss avoidance context—errors and slow responses were punished whereas fast and correct responses resulted in loss omission. In the two-step task (detailed in [67]), participants chose between two first-stage stimuli (spaceships), each transitioning with 80% probability (common transition) to one of two second-stage stimuli (planets) and with 20% probability (rare) to the other. Here, participants again chose between two stimuli (aliens) for potential reward or loss (second-stage feedback).

### Questionnaires

#### UPPS scale

The UPPS Impulsive Behavior Scale (UPPS; [15]; German version: [68]) involves 45 items with four-point Likert scales (from 1 – ‘strongly disagree’ to 4 – ‘strongly agree’) to assess the impulsivity facets negative urgency, lack of premeditation, lack of perseverance, and sensation seeking, all of which have shown good psychometric properties. We used the sum score of the twelve-item negative urgency subscale (*M*±*SD* = 26.3±5.9, range: 14–43; current internal consistency: Cronbach’s α = 0.87), mirroring other healthy samples [68] and closely matching the theoretical range (12–48).

#### OCI-R

The Obsessive-Compulsive Inventory Revised (OCI-R; [61]; German version: [69]) assesses the severity of obsessive-compulsive symptoms (washing, checking, obsessing, neutralizing, ordering, hoarding) with 18 items on a five-point Likert scale (from 0 – ‘not at all’ to 4 – ‘extremely’; theoretical range: 0–72) and has shown good psychometric properties. We used OCI-R sum scores to measure compulsivity (*M*±*SD* = 12.6±9.5, range: 0–46; current internal consistency: Cronbach’s α = 0.91), indicating mild (OCI-R < = 15) compulsivity on average, with *n* = 60 expressing moderate (OCI-R = 16–27) to severe (OCI-R > = 28) scores [70]. Prior, similar research has used OCI-R sum scores to assess compulsivity [71]. However, acknowledging the heterogeneity of OCD phenotypes [72] and the nosological separation of hoarding disorder from OCD, the Supplementary Materials details OCI-R subscales.

### EEG recording and data reduction

We recorded EEG at 500 Hz with 61 Ag/AgCl electrodes using two 32-channel BrainAmp amplifiers (Brain Products GmbH, Munich, Germany) and an EasyCap with equidistant electrode locations (EasyCap GmbH, Herrsching-Breitbrunn, Germany). The setup included reference and ground electrodes next to Fz, two external electrodes below the left and right eye, and one at the lower back for electrocardiography (the latter removed before offline analyses). Impedances were <10 kOhm.

We filtered data offline 0.1–30 Hz. Ocular and cardiac artifacts were manually removed using adaptive mixture independent component analysis (*runamica12* in EEGLAB) and subsequent visual inspection of the temporospatial distribution of components, assisted by the IClabel plug-in for EEGLAB [73]. Epochs ranged from 200 ms before and 700 ms after go stimulus (GNGT and SST) and feedback (MIFLAT, two-step) onset (criteria for excluding trials from EEG analyses are detailed in the Supplementary Task Descriptions). We rejected artifacts defined as improbable data relative to the activity of all electrodes and epochs (within data from each task), excluding epochs containing deviations > 5 SDs of the mean probability distribution (at single-subject level, no interpolation of EEG data). The 200 ms pre-stimulus windows served as baseline. EEG data showed good to excellent reliability (see supplementary material).

### Statistical analysis of EEG data

#### EEG analysis involved two steps

**Fig. 2:**
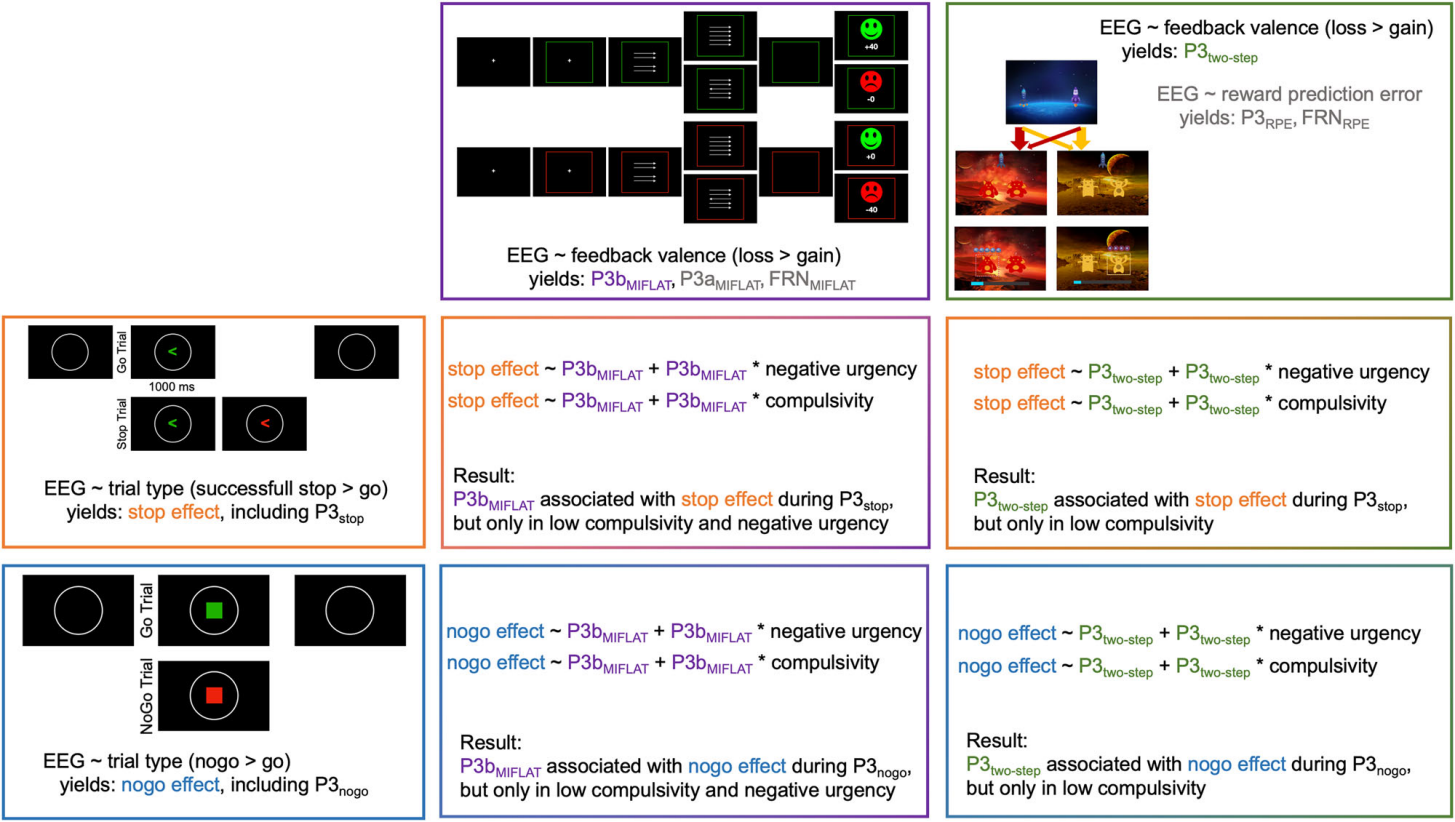
Analysis overview. At the subject-level, we submitted EEG activity from each of the four tasks (blue box: go/nogo task; orange box: stop signal task; purple box: monetary incentive flanker task [MIFLAT]; green box: two-step task) to single-trial regression analyses to establish task effects. Simplified regression formulae are provided with task images; additionally, we name event-related potentials of interest at which task effects were prominently expressed. The main text of this report focuses on EEG activity tied to the P3 obtained from each task (P3b_MIFLAT_, P3t_wo-step_, P3_stop_, P3_nogo_). Additionally, components listed in gray font are described in the supplementary materials. This includes, for the MIFLAT, the feedback-related negativity (FRN_MIFLAT_) and P3a_MIFLAT_ and, from single-trial regressions using the reward prediction error (RPE) as predictor of EEG activity in the two-step task, the FRN_RPE_ and P3_RPE_. The central four boxes, merging the colors of those tasks involved in the respective regression analyses, illustrate analyses at the second level with the regression formula and a brief summary of the result. We used centroparietal EEG activity sensitive to the feedback valence task effects in the MIFLAT and two-step tasks (P3b_MIFLAT_ and P3_two-step_; for quantification details, see Statistical analysis of EEG data: Second-level analyses) as a predictor of the stop effect (trial type task effect in the stop signal task) and the nogo effect (trial type task effect in the go/nogo task) at each electrode and time point. Importantly, these regression models further included interaction terms involving self-reported negative urgency and compulsivity scores to assess moderation by these transdiagnostic risk factors.

First-level analyses aimed to establish the neural signature of trial-wise parameters, i.e. feedback valence (MIFLAT, two-step), successfully stopping a response vs. going (SST), and successfully withholding a response vs. going (GNGT). Second-level analyses served hypothesis testing and took first-level estimates (b-values) to the group level, examining whether the association between feedback-related activity (MIFLAT, two-step) and motor inhibition (GNGT: withholding; SST: stopping) was moderated by compulsivity and negative urgency. We visualized our workflow in Fig. 2. Data and code are available at https://osf.io/qb4jp/.

#### First-level analyses

**Fig. 3:**
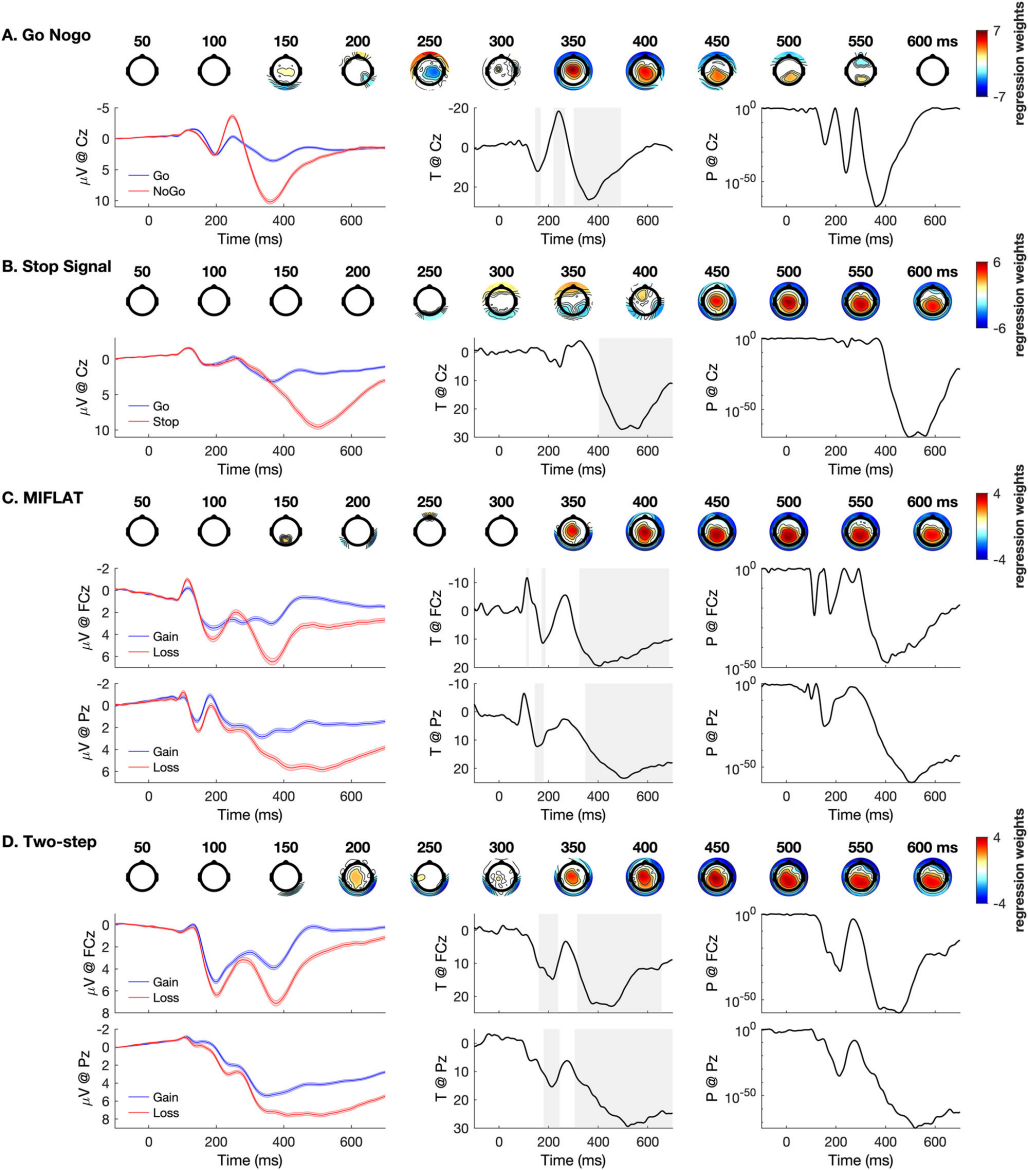
Depiction of first-level regression effects for suppression, stopping, and feedback valence. Effects of the trial type regressors (**A**) nogo vs. go at Cz in the go/nogo task, (**B**) successful stop vs. go at Cz in the stop signal task, (**C**) loss vs. gain at FCz and Pz in the monetary incentive flanker task (MIFLAT), and (**D**) loss vs. gain at Pz in the two-step task are shown. In each figure section, top rows present topographical maps of significant associations between EEG activity and the regressor in question (*t*-values, red: positive, blue: negative, masked at *p* = 10^−20^; critical *p* = 0.015 in (**A**); critical *p* = 0.014 in (**B**); critical *p* = 0.016 in (**C**), critical *p* = 0.014 in (**D**). The lower left, center, and right show original event-related potential waveforms (shades reflect standard error of the mean), trajectories of *t*-values for the regressor at selected electrodes (resulting from one-sample t-tests of single-trial regression weights against zero; gray shades reflect significance at *p* = 10^−20^), and time courses of corresponding *p*-values, respectively.

We used multiple single-trial robust regression [74, 75], which is relatively unbiased by outliers or heteroskedastic distributions, to predict individual EEG data at each electrode and time point (all regression statistics were obtained for the −100–700 ms interval around the locking stimulus) from trial-wise task parameters. Temporal smoothing of EEG data involved averaging activity at each time point with the preceding and succeeding two time points (no spatial smoothing occurred). Individual b-values from these regressions reflect the relationship of EEG activity with each regressor at each electrode and time point, yielding electrode-specific time courses of regression weights. We compared b-values against zero using two-tailed (alpha = 0.025) one-sample t-tests and employing false discovery rate with the Benjamini-Hochberg procedure (FDR; [76]), accounting for the number of all electrodes (*n* = 63) and time points (*n* = 401) to correct for multiple comparisons. Benjamini-Hochberg controls FDR well under the assumption of positively regression dependent data [76], which is reasonable to assume given the temporospatial autocorrelation inherent to EEG recordings. This yielded time-variant topographies of significant relationships between EEG data and task-related predictors (feedback valence, successful stop vs. go, nogo vs. go; see Eqs. 1–3), as shown in Fig. 3.

For the GNGT, trial type (go, nogo; coded as −1 and 1) was the predictor of interest, with the duration of the preceding inter-trial interval as a regressor of no interest:

1 $${\rm{EEG}}={{\rm{\beta }}}_{0}+{{\rm{\beta }}}_{1}\times {trial}\,{type}+{{\rm{\beta }}}_{2}\times {inter}-{trial}\,{interval}+{Error}$$

For the SST, trial type (go, successful stop; coded as −1 and 1) was the predictor of interest, with arrow direction and the duration of the preceding inter-trial interval as regressors of no interest:

2 $${\rm{EEG}}={{\rm{\beta }}}_{0}+{{\rm{\beta }}}_{1}\times {trial}\,{type}+{{\rm{\beta }}}_{2}\times {arrow}\,{direction}+{{\rm{\beta }}}_{3}\times {inter}-{trial}\,{interval}+{Error}$$

For the MIFLAT and two-step task, the GLM included the regressor feedback valence:

3 $${\mathrm{EEG}}={{\rm{\beta }}}_{0}+{{\rm{\beta }}}_{1}\times {feedback}\,{valence}+{Error}$$

In the MIFLAT, feedback valence was positive in the potential gain context and negative in the loss avoidance context (coded as −1 and 1, respectively). For the two-step task, we excluded feedback yielding zero points and defined feedback yielding < zero and > zero points as losses (coded as 1), gains (coded as −1), respectively. Note that this analysis of two-step data went beyond our preregistered analyses and should therefore be considered exploratory. The supplementary material details the reward prediction error as a parametric predictor of trial-wise EEG activity.

We report statistics (betas, *t*-values, *p*-values, degrees of freedom, confidence intervals, Hedge’s *g* as effect size, critical *p*-value from FDR correction) for time points at electrodes showing the strongest effects. We searched for peaks in regression effects associated with the P3 in the GNGT (P3_nogo_) at central electrodes > 300 ms after target onset, the P3 in the SST (P3_stop_) at centroparietal electrodes > 400 ms after go onset, the feedback-related P3b in the MIFLAT (P3b_MIFLAT_) as well as the feedback-related P3 in the two-step task (P3_two-step_) at centroparietal electrodes > 350 ms after feedback onset (detailed in Table 1), based on prior results [52]. Otherwise, we selected peaks based on statistics and treated all t-tests as independent (no temporal or spatial clustering occurred). Analyses were conducted with MATLAB 2023a (MathWorks) and EEGLAB 2020 [77].

**Table 1:** Peak Statistics for the Effects of Withholding (Go / Nogo Task), Stopping (Stop Signal Task), and Feedback Valence (Monetary Incentive Flanker Task and Two-Step Task) on EEG Activity Related to the P3.

	Electrode	Time (ms)	*t*^a^	*p* (critical *p*)	Confidence Interval^b^	*g*
Go / Nogo Task						
P3_nogo_	FC1	366	27.74	<10^−70^ (0.015)	5.70, 6.80	1.93
Stop Signal Task						
P3_stop_	P2	564	30.97	<10^−78^ (0.014)	4.47, 5.25	2.15
MIFLAT						
P3b_MIFLAT_	Pz	504	23.49	<10^−59^ (0.016)	3.49, 4.29	1.63
Two-Step Task						
P3_two-step_	CP1	448	30.93	<10^−78^ (0.018)	3.83, 4.47	2.16

Electrode and Time (ms) indicate spatial and temporal peaks of the effect. Crit *p*, significance threshold from false discovery rate correction; *g*, Hedge’s *g*, *MIFLAT* monetary incentive flanker task.

^a^*df* = 204.

^b^100 x (1 – critical *p*) %.

Note that we previously published and investigated stop signal and nogo task as well as reward prediction error (two-step task) main effects from the full sample of the overall study in terms of relationships with impulsivity and compulsivity [62, 63, 67]. In this report, main effects only serve as prerequisites for hypothesis testing and are reiterated specifically for the current sample. However, for completeness, we present main effects of compulsivity and negative urgency on task effects within the current sample in the supplementary materials.

#### Second-level analyses

To examine the effect of compulsivity, we set up GLMs to predict nogo (GNGT) and stop signal (SST) effects as captured in the following general formula:

4 $${\rm{First}}-{\rm{level\; b}}={{\rm{\beta }}}_{0}+{{\rm{\beta }}}_{1}\times {feedback}\,{effect}+{{\rm{\beta }}}_{2}\times {feedback}\,{effect}* {compulsivity}+{Error}$$

Here, first-level b refers to the effect of the trial type regressor in the GNGT or SST at each electrode and time point. As feedback effect, we used the mean of first-level b-values of the feedback valence regressor in the MIFLAT, corresponding to the P3b_MIFLAT_ (Pz, 500–520 ms), and the feedback valence regressor in the two-step task, corresponding to the P3_two-step_ (CP1, 440–460 ms; in the supplementary materials, we present effects of EEG activity associated with other feedback-related components). For the interaction term, variables were demeaned and then multiplied.

Thus, to predict the stop signal effect, we regressed b-values of the SST trial type regressor at each electrode and time point on (a) mean first-level b-values representing the P3b_MIFLAT_ and on their interaction with compulsivity, and (b) mean first-level b-values representing the P3_two-step_ and on their interaction with compulsivity (masked at *p* = 0.010). We applied the same procedure to predict the nogo effect (GNGT trial type regressor). This amounted to four sets of second-level regressions: P3b_MIFLAT_ effects predicting stop signal (SST) and nogo (GNGT) effects; P3_two-step_ effects predicting stop signal (SST) and nogo (GNGT) effects.

We followed the same second-level procedure for negative urgency (while our pre-registered hypothesis was specific to negative urgency, we explore other UPPS subscales in the supplementary materials):

5 $${\rm{First}}-{\rm{level\; b}}={{\rm{\beta }}}_{0}+{{\rm{\beta }}}_{1}\times {feedback}\,{effect}+{{\rm{\beta }}}_{2}\times {feedback}\,{effect}* {negative}\,{urgency}+{Error}$$

We explored significant moderation (interactions with compulsivity and negative urgency investigating Hypothesis A) using Johnson-Neyman analyses (JN; [78]), to find intervals of the moderator in which the predictor was significantly vs. non-significantly associated with the dependent variable (threshold: *p* = 0.01).

## Results

### Compulsivity moderates the relationship of stopping and withholding with feedback processing from both feedback tasks

#### Stop Signal Task

**Fig. 4:**
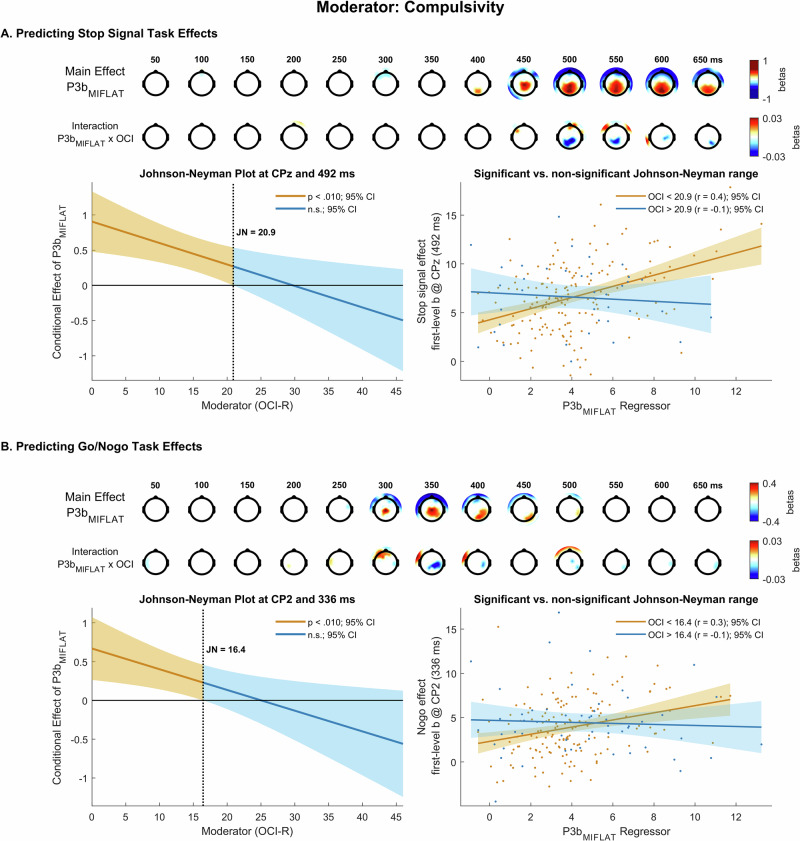
Moderation effects of compulsivity. The (first-level) Monetary Incentive Flanker Task P3b (P3b_MIFLAT_) and its interaction with compulsivity (OCI; Obsessive-Compulsive Inventory) predict (first-level) stop signal effects in the stop signal task (**A**) and (first-level) nogo effects in the go/nogo task (**B**). In each figure section, the two top rows present topographical maps of significant associations of the P3b_MIFLAT_ (main effect; first row) as well as the interaction between P3b_MIFLAT_ and OCI scores (interaction effect; second row) with first-level stop signal (**A**) and nogo (**B**) effects. The lower left panels show Johnson-Neyman (JN) plots depicting simple slopes of the main effect as a function of score on the moderator, with vertical lines indicating JN points at which the main effect switches from significant (orange) to non-significant (blue). The lower right panels show scatterplots, contrasting OCI groups scoring below and above the JN point, of the relationship between the P3b_MIFLAT_ regressor as entered into the model (Eq. 5) and first-level b-values for the stop signal (**A**) and nogo (**B**) effect at a parietal electrode and time point where the interaction effect of the P3b_MIFLAT_ with compulsivity was maximal. All topographical maps display regression weights (betas) from between-subjects analyses, red: positive, blue: negative, masked at *p* = 0.010.

The P3b_MIFLAT_ regressor significantly and positively predicted the first-level stop signal effect (EEG activity sensitive to the contrast successful stop > go) in the P3 (Pz peak at 594 ms, β = 0.54, 99% CI [0.35 0.74], *t*(204) = 7. 73, *p* = 4.21 ×10^−12^; Fig. 4A). Additionally, the interaction term OCI-R x P3b_MIFLAT_ was significant at centroparietal sites (CPz peak at 492 ms, β = −0.03, 99% CI [−0.05 −0.008], *t*(204) = −3.52, *p* = 5.32 ×10^−4^), i.e. during the peak of the P3_stop_. Contrary to our hypothesis A, the centroparietal relationship between P3b_MIFLAT_ and P3_stop_ was significant for mild to moderate (JN point: OCI-R < 20.9 at CPz and 492 ms) but not moderate to severe OCI-R scores.

**Fig. 5:**
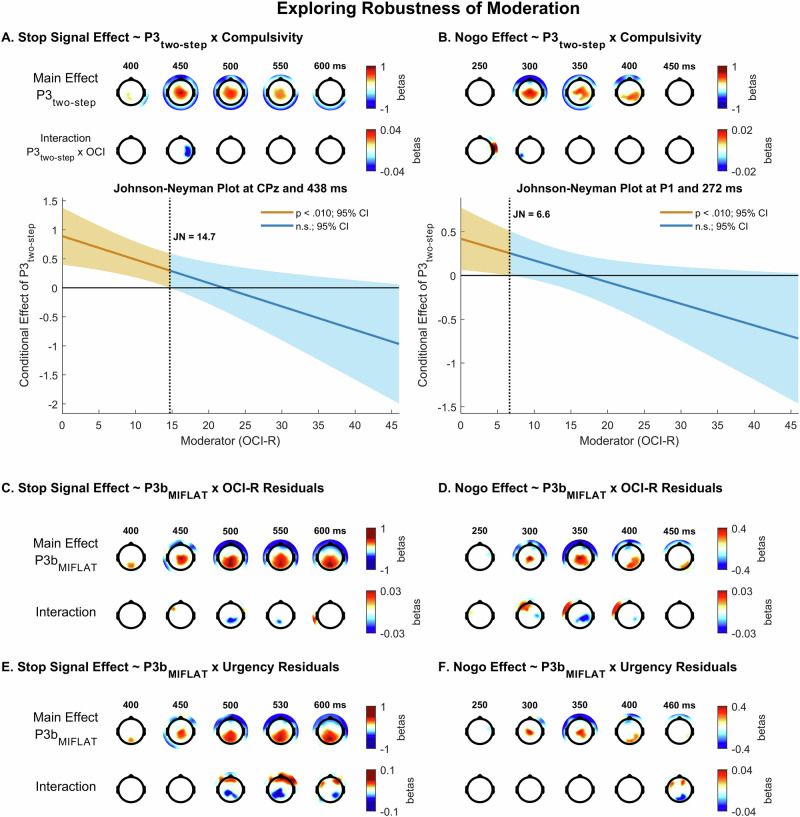
Robustness of moderation by compulsivity. Moderation effects of compulsivity (OCI; Obsessive-Compulsive Inventory-Revised) in predicting (first-level) stop signal effects in the stop signal task (**A**) and nogo effects in the go/nogo task (**B**) remain robust when considering interactions between the P3 from the two-step task (P3_two-step_) and compulsivity. Compulsivity after controlling for variance related to negative urgency (OCI-R residuals) remains a significant moderator of the effect of P3b_MIFLAT_ on stop signal (**C**) and nogo (**D**) effects. The same was observed for negative urgency after controlling for compulsivity (urgency residuals; (**E**–**F**)). In each figure section, the two top rows present topographical maps of significant main effects (first row) as well as interaction effects (second row) on first-level stop signal or nogo effects. In (**A**) and (**B**), the lower panels show Johnson-Neyman (JN) plots depicting simple slopes of the main effect as a function of score on the moderator, with vertical lines indicating JN points at which the main effect switches from significant (orange) to non-significant (blue). All topographical maps display regression weights (betas) from between-subjects analyses, red: positive, blue: negative, masked at *p* = 0.010.

The P3_two-step_ regressor significantly and positively predicted the stop signal effect in the P3 (Cz peak at 450 ms, β = 0.91, 99% CI [0.45 0.1.37], *t*(204) = 5.18, *p* = 5.89 ×10^−7^; see Fig. 5A). Additionally, the interaction term OCI-R x P3_two-step_ was significant at centroparietal sites (CP4 peak at 438 ms, β = −0.04, 99% CI [−0.07 −0.01], *t*(204) = −3.52, *p* = 5.65 ×10^−4^; see Fig. 5A), i.e. during the early P3_stop_. The centroparietal relationship between P3_two-step_ and P3_stop_ was significant for mild (JN point: OCI-R < 14.7 at CP4 and 438 ms) but not moderate to severe OCI-R scores.

#### Go / Nogo Task

The P3b_MIFLAT_ regressor significantly and positively predicted the nogo effect (EEG activity sensitive to the contrast nogo > go) associated with the P3_nogo_ (Cz peak at 324 ms, β = 0.48, 99% CI [0.15 0.80], *t*(204) = 4.16, *p* = 2.02 ×10^−4^; Fig. 4B). Additionally, the interaction term OCI-R x P3b_MIFLAT_ was significant at centroparietal sites (CP2 peak at 336 ms, β = −0.03, 99% CI [−0.05 −0.006], *t*(204) = −4.09, *p* = 0.001), i.e. during the peak of the P3_nogo_. The centroparietal relationship between P3b_MIFLAT_ and P3_nogo_ was significant for mild but not moderate to severe OCI-R scores (JN: OCI-R < 16.4 at CP2), again contrasting with our hypothesis A.

Further, the P3_two-step_ regressor significantly and positively predicted the nogo effect in the P3 (Cz peak at 292 ms, β = 0.68, 99% CI [0.58 0.77], *t*(204) = 4.60, *p* = 1.54 ×10^−5^). The interaction term OCI-R x P3_two-step_ was significant at parietal sites (P1 peak at 272 ms, β = −0.02, 99% CI [−0.03 −0.017], *t*(204) = −3.01, *p* = 0.003; Fig. 5B). The parietal relationship between P3b _two-step_ and P3_nogo_ was significant for only the lowest OCI-R scores (JN point: OCI-R < 6.6 at P1 and 272 ms). The main effect, and the interaction in particular, occurred during the earliest ramp-up of the nogo effect and should thus be treated with caution.

### Negative urgency moderates the relationship of stopping and withholding with feedback processing in the MIFLAT

#### Stop Signal Task

**Fig. 6:**
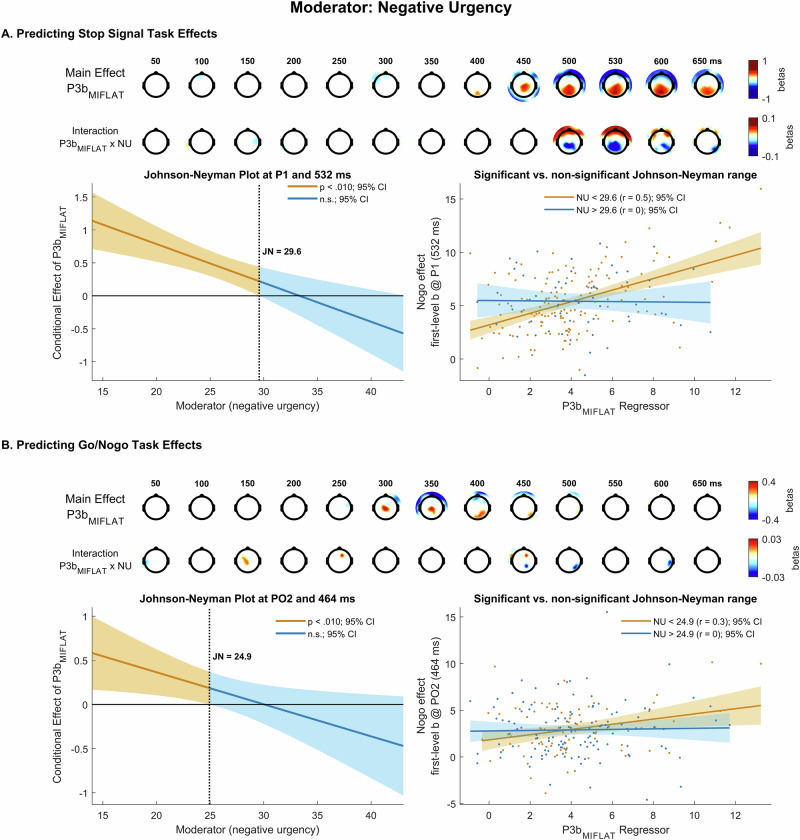
Moderation effects of negative urgency. The (first-level) Monetary Incentive Flanker Task (MIFLAT) P3b (P3b_MIFLAT_) and its interaction with negative urgency (NU) predict (first-level) stopping effects in the stop signal task (**A**) and (first-level) nogo effects in the go/nogo task (**B**). In each figure section, the two top rows present topographical maps of significant associations of the P3b_MIFLAT_ (main effect; first row) as well as the interaction between P3b_MIFLAT_ and NU scores (interaction effect; second row) with first-level stop signal (**A**) and nogo (**B**) effects. The lower left panels show Johnson-Neyman (JN) plots depicting simple slopes of the main effect as a function of score on the moderator, with vertical lines indicating JN points at which the main effect switches from significant (orange) to non-significant (blue). The lower right panels show scatterplots, contrasting NU groups scoring below and above the JN point, of the relationship between the P3b_MIFLAT_ regressor as entered into the model (Eq. 5) and first-level b-values for the stop signal (**A**) and nogo (**B**) effect at a parietal electrode and time point where the interaction effect of the P3b_MIFLAT_ with NU was maximal. All topographical maps display regression weights (betas) from between-subjects analyses, red: positive, blue: negative, masked at *p* = 0.010.

Similar to OCI-R, the interaction term negative urgency x P3b_MIFLAT_ emerged significant at parietal sites (P1 peak at 532 ms, β = −0.06, 99% CI [−0.09 −0.03], *t*(204) = −4.76, *p* = 3.64 ×10^−6^; Fig. 6A). The parietal relationship between P3b_MIFLAT_ and P3_stop_ was significant for low but not high urgency scores (JN: urgency < 29.6 at P1 and 532 ms), contrasting hypothesis A.

The interaction term negative urgency x P3_two-step_ did not modulate the relationship between the P3_two-step_ and the stop signal effect (P10 peak at 608 ms, β = −0.08, 99% CI [−0.014 −0.02], *t*(204) = −3.85, *p* = 0.001).

#### Go / Nogo Task

The interaction term negative urgency x P3b_MIFLAT_ was also significant for the nogo effect (PO2 peak at 464 ms, β = −0.04, 99% CI [−0.07 0.006], *t*(204) = −4.30, *p* = 0.003; Fig. 6B). The parietal relationship between P3b_MIFLAT_ and P3_nogo_ was significantly positive for low but not high urgency scores (JN: urgency < 24.9 at PO2 and 464 ms), contrasting hypothesis A.

Regarding the P3_two-step_, the interaction term negative urgency x P3_two-step_ was not significant (IO1 peak at 430 ms, β = −0.04, 99% CI [−0.06 −0.03], *t*(204) = −2.28, *p* = 0.084).

### Exploring the unique influences of compulsivity and negative urgency

Negative urgency and OCI-R scores showed a weak positive relationship, *r*(203) = 0.18, *p* = 0.010. Given the similar moderating influence of compulsivity and negative urgency on the relationship between the P3b_MIFLAT_ and P3_stop_ as well as P3_nogo_, we went beyond our preregistered analyses and examined whether these effects were driven by shared or unique variance. In order to do so, we first saved raw residuals from regressing OCI-R and negative urgency scores on each other, i.e. partial variance unique to each construct. Second, we repeated the second-level regression models predicting the task effect in the SST and GNGT data with these residual scores in the interaction term (Fig. 5C-F).

#### Stop Signal Task

We observed significant interactions for residualized OCI-R scores (*SD* = 9.3, range: −14.6 – 32.2; main effect: Pz peak at 596 ms, β = 0.54, 99% CI [0.34 0.73], *t*(204) = 7.24, *p* = 9.43 ×10^−12^; interaction term: P1 peak at 530 ms, β = −0.03, 99% CI [−0.05 −0.01], *t*(204) = −3.84, *p* = 1.63 ×10^−4^; JN point: OCI-R_resid_ = 8.1) and residualized negative urgency scores (SD = 5.8, range: −12.4 – 16.5; main effect: Pz peak at 602 ms, β = 0.50, 99% CI [0.32 0.69], *t*(204) = 7.17, *p* = 2.19 ×10^−11^; interaction term: P1 peak at 530 ms, β = −0.05, 99% CI [−0.09 0.02], *t*(204) = −4.06, *p* = 7.76 ×10^−5^; JN point: urgency_resid_ = 3.4), indicating moderating effects unique to each construct.

#### Go/Nogo task

We found significant interactions for residualized OCI-R scores (main effect: Cz peak at 322 ms, β = 0.49, 99% CI [0.16 0.81], *t*(204) = 4.19, *p* = 1.52 ×10^−4^; interaction term: CP2 peak at 336 ms, β = −0.03, 99% CI [−0.05 −0.01], *t*(204) = −5.47, *p* = 6.87 ×10^−4^. JN point: OCI-R_resid_ = 3.9) and residualized urgency scores, though prominently later than the main effect (main effect: Cz peak at 318 ms, β = 0.44, 99% CI [0.13 0.75], *t*(204) = 3.66, *p* = 3.70 ×10^−4^; interaction term: PO2 peak at 466 ms, β = −0.04, 99% CI [−0.07 −0.004], *t*(204) = −4.26, *p* = 0.004. JN point: urgency_resid_ = −1.8).

## Discussion

We investigated whether compulsivity and negative urgency moderated the relationship between neural correlates of feedback processing and motor inhibition. Both compulsivity and negative urgency significantly moderated the relationship between loss sensitivity (MIFLAT) and both withholding and stopping. For compulsivity, moderation also emerged in the two-step task. However, in contrast to our hypothesis, high compulsivity and negative urgency were related to decreased associations between valence and inhibitory processing. Moderation by compulsivity remained significant when controlling for negative urgency, and vice versa.

Our preregistered hypotheses were rooted in imbalance frameworks of impulsive-compulsive psychopathology positing hyperactivity in salience and emotion networks accompanied by impaired prefrontal functioning [43]. Specifically, hypotheses were informed by previous within-subjects brain-brain indices of imbalance in bingeing behaviors. To reiterate, low inhibitory activity was linked to high reward sensitivity (gain > loss contrast) in binge-drinkers [51] and low loss sensitivity (loss > gain contrast) in high binge-watchers [52], but not the respective control groups. Reusing tasks and analyses from our previous work, we assumed in analogy that high compulsivity and negative urgency individuals should show a stronger relationship between inhibitory activity and loss sensitivity. We were therefore surprised to observe the opposite.

However, prior results were interpreted in light of a zero-relationship in the respective control groups. In analogy, the lower end of the compulsivity and negative urgency spectra might resemble control groups with few maladaptive behaviors. Given that we observed a positive relationship between feedback and inhibitory processing in low compulsivity and low negative urgency individuals, we reconsider how imbalance is reflected in our data. Results indicate that high loss sensitivity is related to high inhibitory activity when compulsivity and urgency scores are low. Individuals characterized by low compulsivity and urgency should be able to compensate affective urges with high inhibitory activity, striking a balance that shields the pursuit of long-term goals from short-term emotional interference. In contrast, this relationship is significantly reduced in high compulsivity and urgency individuals, indicating that, when they encounter greater losses, they may be less able to access the inhibitory resources necessary to regulate affective reactivity. With the systems out of balance, goal-directed behavior may be more likely to be compromised by arousal. This aligns with highly compulsive phenotypes, where distress-related urges compel individuals to repetitive behaviors often incompatible with overall goals [3, 7]. Similarly, in negative urgency, negative affect can elicit disinhibited behaviors which often result in undesirable consequences [16, 79].

These observations at subclinical levels of compulsivity and negative urgency have important implications for dual-systems frameworks of impulsive-compulsive psychopathology. Given the transdiagnostic risk associated with these constructs [8, 19, 80], neural imbalance may serve as a potential vulnerability marker. This is important to consider because such imbalance need not preexist but could develop through various mechanisms, such as learning processes that change the salience of drug cues and rewards or withdrawal-related dysphoria over prolonged substance use [81, 82], neurotoxic effects of substances [83], or the progressive manifestation of compulsive urges and rituals [84, 85]. Further, the notion of imbalance as a vulnerability is consistent with prospective evidence within isolated psychological domains, particularly regarding the predictive value of diminished inhibitory brain activity in addictions [86]. Investigating imbalance in a subclinical sample is a strength of our study because alterations in clinical groups could be confounded by factors such as prolonged substance exposure.

It should be stressed that imbalance frameworks, while supported by meta-analyses [34, 35, 38, 39], largely draw on isolated investigations of networks for control and salience or emotion. In contrast, our study captured brain activity from both domains within each participant. Main effects of negative urgency and compulsivity on task effects (see supplementary materials) indicated that negative urgency, but not compulsivity, was associated with lower P3_nogo_ and P3_stop_. Similar distinctions emerged for feedback processing. These differences starkly contrast with the similarities revealed by between-domain measures, suggesting that focusing on isolated hyper- and hypoactivities to assess imbalance may potentially obscure relevant information. The current results therefore indicate that imbalance frameworks of impulsive-compulsive behaviors should be assessed through between-domain associations.

Results for compulsivity and negative urgency robustly showed moderation across motor inhibition tasks. Withholding involves a psychophysiological cascade of low complexity from early sensory to attentional to motor control processes that facilitate suppressing a response [65]. Stopping relies on a very early proactive control component that influences sensory and attentional preparation for the stop signal and subsequent reflexive implementation of motor inhibition. This suggests that imbalance in compulsivity and urgency is sensitive to a range of inhibitory demands, in which even low complexity can challenge control over compulsive urges and affective impulses. This may render a broad set of circumstances with inhibitory requirements relevant to the development of impulsive-compulsive psychopathology.

It is intriguing that imbalance in each construct remained when parsing out shared variance. Consistently, compulsivity and impulsivity are assumed to overlap substantially and have unique aspects [6, 7]. They reflect dimensional phenotypes or transdiagnostic markers which, due to their behavioral and neurobiological commonalities, both contribute to psychopathology. The current brain-brain imbalance presumably captures variance from the impulsive-compulsive core, disinhibition involved in coping with emotional or stress-related discomfort, as well as compulsivity-specific rigidity and urgency-specific rashness [8, 87].

It is a limitation that the current sample reported fewer mental health problems than the general population [88], which also were not professionally verified. A less selective sample could have revealed the relevance of concrete problematic behaviors like drug use. Assessing compulsivity with the OCI-R sum score does not sufficiently acknowledge the heterogeneity of obsessive-compulsive phenotypes [72].

Advancing our understanding of neural imbalance in psychopathology requires investigating interrelationships between systems within individuals and clarifying their role in transdiagnostic risk factors. Using multiple tasks, we provide evidence for an imbalance between feedback processing and motor inhibition in both high compulsivity and negative urgency, suggesting a potential inability to compensate sensitivity to motivationally relevant stimuli with sufficient motor inhibition. Longitudinal or family-risk studies could test whether imbalance confers risk for impulsive-compulsive psychopathology.

## Supplementary information

Supplemental Material for Neural Imbalance in Compulsivity and Negative Urgency

## Data Availability

Data and Code are available at https://osf.io/qb4jp/.
