# Supplementary material for: Neural imbalance between feedback sensitivity and motor inhibition in compulsivity and negative urgency
Source: Transl Psychiatry. 2026 May 15;16:248. doi: 10.1038/s41398-026-04098-z (PMC13179386; doi:10.1038/s41398-026-04098-z)
Supplement: Supplementary file 1 — Supplemental Material for Neural Imbalance in Compulsivity and Negative Urgency [file 41398_2026_4098_MOESM1_ESM.docx]

Supplementary materials for the article “Neural imbalance between feedback sensitivity and motor inhibition in compulsivity and negative urgency”

These supplementary materials present methods and results regarding two further pre-registered hypotheses as well as exploratory analyses, detailed sample characteristics, and reliability estimates.

**Supplementary Sample Information**

We assessed inclusion (German native speakers, normal or corrected-to-normal vision; age 18 - 45) and exclusion (history of neurological disorder or head trauma; lifetime bipolar disorder, borderline personality disorder, psychotic episodes, or severe alcohol use disorder [>5 DSM-5 criteria]; present eating disorders or severe episodes of major depression; lifetime use of any illicit drug more than twice per year per substance; lifetime cannabis use more than twice per month; use of psychotropic substances within the past three months) criteria via online survey and telephone interviews. For the current report, we only included n = 205 participants from the original n = 252 sample for whom data from all four tasks were available to examine a homogenous sample across analyses: We excluded *n* = 8 due to technical recording errors on at least one of the tasks, *n* = 17 because they had fewer than two valid blocks in the SST (see Supplementary Task Description below for definition of valid blocks), *n* = 2 because their response times on unsuccessful stop trials was longer than on go trials in the SST, prohibiting estimation of the stop signal reaction time as per Verbruggen et al. (^1^; while the SSRT was not a variable of interest in this analysis, this ensured a homogeneous sample in terms of underlying assumptions regarding the horse-race model of the SST), *n* = 9 due to insufficient performance in the MIFLAT (> 40% errors), and *n* = 11 due to random choice behavior in the two-step task, i.e. their probability to repeat the last first-stage choice (stay probability) was not positively associated with the reward or reward*transition interaction of the last trial in a logistic regression model. In the online survey, asking to self-report any mental disorders, we recorded *n* = 8 with depression, *n* = 5 with any anxiety disorder, n = 5 with depression and an anxiety disorder, *n* = 1 with any eating disorder, n = 1 with depression and an eating disorder, *n* = 1 with posttraumatic stress disorder, n = 1 with depression and posttraumatic stress disorder, *n* = 1 with an anxiety and obsessive-compulsive disorder, *n* = 1 with a somatoform disorder.

**Supplementary Task Descriptions**

*Go/nogo task*. Participants viewed a white circle on a gray background for 200-500 ms (see Figure 1). Then, a green square (go signal, 75 % of trials), indicating to respond as quickly as possible with the dominant index finger or a red square (nogo signal, 25 % of trials), indicating to withhold a response, occurred for 500 ms, followed by inter-trial intervals (400 - 1000 ms). A maximum of five go trials separated up to two subsequent nogo trials. Participants performed two blocks with 128 trials.

We excluded trials with early (< 100 ms) or late (> 600 ms) responses and go trials with omitted responses from analyses.

*Stop signal task*. Participants viewed a white circle on a gray background for 300-500 ms. Then, a green right- or left-pointing arrow (go signal, 1000 ms), asked to indicate its direction as quickly and accurately as possible with the index fingers. On stop trials (25%), the arrow turned red after the stop signal delay (SSD), asking to cancel the already initiated response. To ensure ~50% stopping performance, the task used four starting SSDs (100, 150, 200, and 250 ms), each active on 25% of stop trials and adapted by their own algorithm (^2^) such that they increased or decreased by 50 ms (lower and upper limits: 50 and 350 ms) after successful or failed stops, respectively. Go and stop trials occurred in pseudorandom order within the constraints that the first three trials were go trials, left and right arrows occurred equally often within go and stop trials, and stop trials and arrow direction did not repeat more than twice. Inter-trial intervals ranged between 400-800 ms. After each block (128 trials), inhibition rates were evaluated but not presented to participants, and participants received feedback to either respond faster (inhibition rate > 60%) or to respond more accurately (inhibition rate < 40%). Training carefully did not mention the ~50% stopping criterion. The task stopped after two valid blocks (stopping accuracies between 40 and 60%) or a maximum of four blocks. Invalid blocks were not analyzed and all participants included in the analyses completed two valid blocks.

We excluded trials with early (< 80 ms) or late (> 600 ms) responses, go trials with omitted or erroneous responses as well as failed stop trials from analyses.

Feedback tasks

*Monetary incentive flanker task (MIFLAT).* In this modified flanker task (^3^), a green or red frame (500 ms) indicated a potential gain or loss avoidance context (50% of trials each), respectively. Four vertical flanker arrows appeared and after 100 ms the central target arrow was presented for 30 ms. The directions of the target and flanker arrows were compatible on half of the trials, and incompatible (pointing in opposite directions) in the remaining trials. Context, compatibility, and target direction were presented in pseudo-random order and balanced between conditions. Participants were asked to press the left or right index finger according to the direction of the target arrow as quickly and accurately as possible, followed by performance feedback (800 ms) 600 ms after the response. Here, we used an adaptive response deadline based on performance and reaction time to obtain a rate of 20% negative feedbacks after correct responses within each context. In the potential gain context, fast (below the deadline) and correct responses were rewarded (40 points; happy green emoji) and errors and slow responses resulted in reward omission (0 points; sad red emoji; gain omission trials). In the loss avoidance condition, errors and slow (exceeding the deadline) responses were punished (minus 40 points; sad red emoji) and fast and correct responses resulted in loss omission (0 points; happy green emoji; loss omission trials). Participants could earn a bonus of up to 5 EUR. The task included 640 trials.

We excluded trials with early (< 150 ms) or late (> 600 ms), erroneous, and omitted responses, as well as loss and gain omission trials from analyses.

*Two-step task*. The modified two-step task involved two subsequent stages of decision making. Participants chose between two first-stage stimuli (spaceships; until response or a maximum of 2000 ms) with their index fingers, their selection was marked for 500-800 ms, and they then transitioned to one of two second-stage stimuli (planets). Each first-stage stimulus transitioned with 80% probability (common transition) to one of the two second-stage stimuli and with 20% probability (rare) to the other. In the second stage, participants encountered a pair of stimuli (aliens; until response or a maximum of 2000 ms) specific to each planet and were asked to choose one, which, in turn, added or subtracted points from their total (in the story, aliens were asked to mine and hand out space treasure; feedback was displayed for 1000 ms). Second-stage rewards adhered to a random walk with reflective bounds at +5 (space treasure) and -4 (anti-matter) points. Inter-trial intervals (300-800 ms) showed a black screen. Participants were asked to win as many points as possible, receiving a respective bonus of up to 5 EUR after the task. During training, participants learned that each spaceship had a fixed preference for one planet and that the outcome of each alien would change over time. The task encompassed four blocks of 125 trials each.

We excluded trials with early (< 100 ms) responses and rare transition trials from analyses.

**First Supplementary Hypothesis**

Prior work assessed feedback sensitivity relatively crudely as neural correlates of processing losses vs. gains (^4,5^). However, ample evidence suggests altered reinforcement learning in impulsive-compulsive symptoms (^6^). In reinforcement learning, prediction errors capture deviations of received feedback from feedback that was expected based on prior learning. Future expectancies can then be updated according to the direction and magnitude of the prediction error, i.e. how much better or worse received feedback turned out to be. Thus, prediction errors provide a nuanced, learning-related aspect of feedback sensitivity. We hypothesized that feedback-inhibition relationships based on the crude gain vs. loss contrast in the MIFLAT generalize to feedback processing sensitive to the signed reward prediction error (RPE).

Methods

We first established the neural signature of the trial-wise parametric prediction error. Specifics regarding the computational modeling of choice behavior in the current sample are detailed elsewhere (^7^). We used the following GLM for the feedback-locked EEG:

EEG = β_0_ + β_1_ x reward *prediction error* + *Error* (S1)

As shown in Fig. S1A, we observed a parietal reward prediction error P3 (P3_RPE_; CP5 peak at 386 ms, *t*(204) = 21.09, *p* < 10^-52^, critical *p* = .014, 98.59% CI [1.75, 2.22], *g* = 1.47) in the two-step task. Therefore, as two-step reward prediction error effect in second-level analyses employing Equations (5-7), we used the mean of first-level b-values of the reward prediction error regressor in the two-step task, corresponding to the FRN_RPE_ (FCz, 256-266 ms; for the second supplementary hypothesis) and P3_RPE_ (CP5, 380-390 ms; for the first supplementary hypothesis) to compute the second-level GLM. Based on Equation (5), to predict the stop signal effect, we regressed b-values of the SST trial type regressor at each electrode and time point on mean first-level b-values representing the reward prediction error on the P3_RPE_ and on their interaction with compulsivity (masked at *p* = .010). We applied the same procedure to predict the nogo effect (GNGT trial type regressor). We then repeated the same second-level procedure for negative urgency, based on Equation (6).

Results

Neither Compulsivity nor Negative Urgency Moderate the Relationship of Stopping and Withholding with Reward Prediction Error Processing

*Stop Signal Task.* Whereas the P3_RPE_ regressor significantly and positively predicted the stop signal effect associated with the stop signal P3_stop_ (FCz peak at 462 ms, β = 0.44, 99% CI [0.11 0.77], *t*(204) = 4.09, *p* = 8.11 x 10^-4^; Fig. 5), this was not modulated by the interaction with compulsivity (IO1 peak at 458 ms, β = -0.04, 99% CI [-0.09 0.01], *t*(204) = -3.44, *p* = .049). The same was true for the interaction with negative urgency (O2 peak at 664 ms, β = -0.06, 99% CI [-0.10 -0.02], *t*(204) = -3.70, *p* = 2.94 x 10^-4^; main effect: Cz peak at 446 ms, β = 0.43, 99% CI [0.05 0.82], *t*(204) = 4.06, *p* = .004).

*Go / Nogo Task.* Whereas the P3_RPE_ regressor significantly and positively predicted the nogo effect associated with the P3_nogo_ (FCz peak at 340 ms, β = 0.52, 99% CI [0.18 0.86], *t*(204) = 3.99, *p* = 1.13 x 10^-4^; Fig. 5), this was not modulated by the interaction with compulsivity (IO2 peak at 364 ms, β = -0.04, 99% CI [-0.8 0.002], *t*(204) = -3.90, *p* = .016). The interaction with negative urgency (P10 peak at 216 ms, β = -0.05, 99% CI [-0.8 -0.02], *t*(204) = -4.13, *p* = 9.72 x 10^-5^) did not modulate the main effect (FCz peak at 340 ms, β = 0.51, 99% CI [0.17 0.84], *t*(204) = 4.13, *p* = 1.31 x 10^-4^).


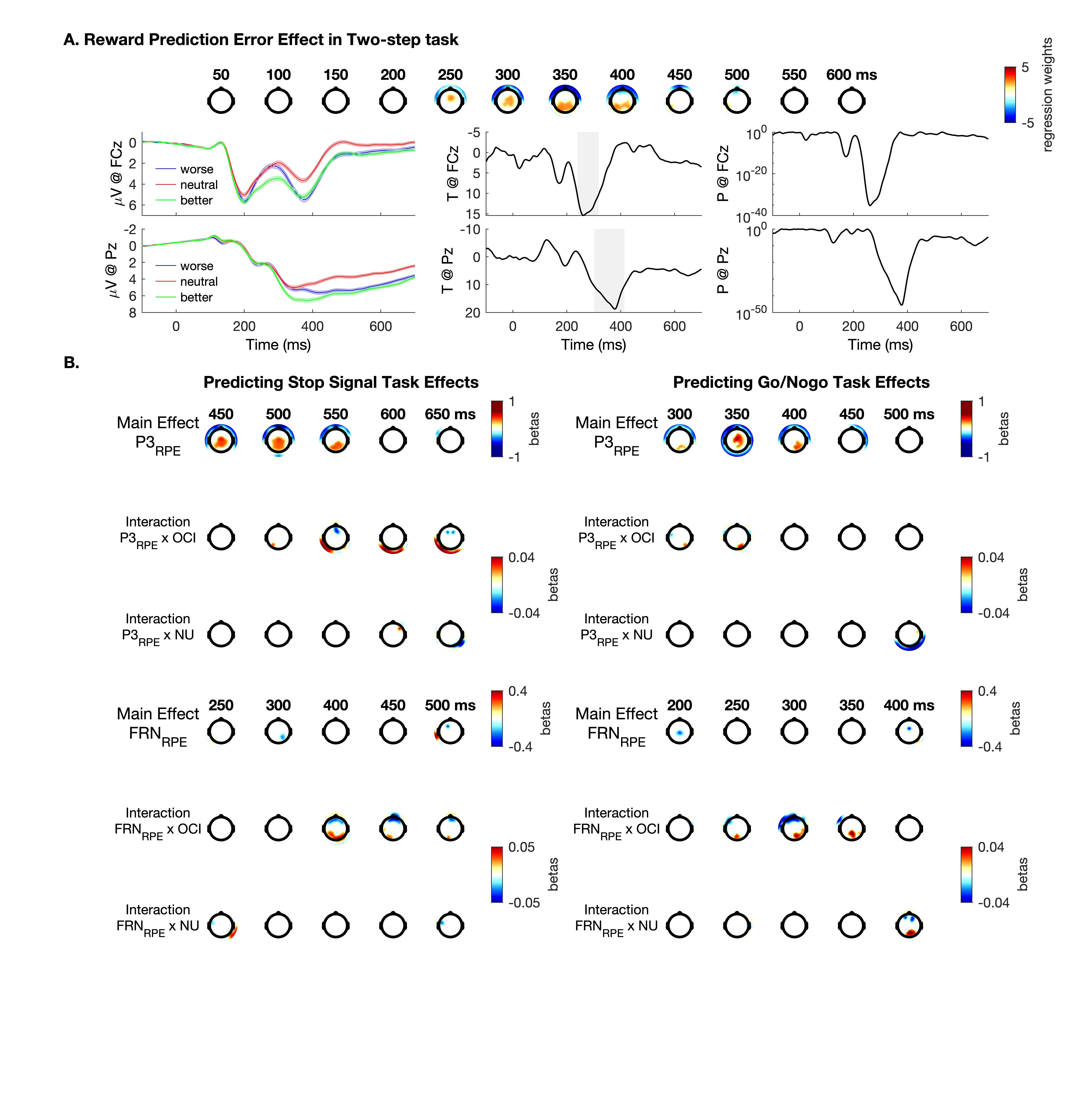


Figure S1. Depiction of the first-level reward prediction error signal and its second-level relationship with motor inhibition. (A) Trial-wise parametric reward prediction error EEG signal at FCz and Pz. The top row presents topographical maps of significant associations between EEG activity and reward prediction error (b-values, red: positive, blue: negative, masked at *p* = 10^-20^; critical *p* = .014). The lower left, center, and right show original event-related potential waveforms (shades reflect standard error of the mean), trajectories of b-values for the regressor at selected electrodes (resulting from one-sample t-tests of single-trial regression weights against zero; gray shades reflect significance at *p* = 10^-20^), and time courses of corresponding *p*-values, respectively. (B) The (first-level) reward prediction error P3 (P3_RPE_), but neither the (first-level) reward prediction error feedback-related negativity (FRN_RPE_) nor interactions with compulsivity (OCI-R; Obsessive-Compulsive Inventory- Revised) or negative urgency (NU), predict (first-level) stop signal effects in the stop signal task (left) and (first-level) nogo effects in the go/nogo task (right). Topographical maps in the top row present significant associations (regression weights [b-values] from between-subjects analyses, red: positive, blue: negative, masked at *p* = 0.010) of the P3_RPE_ (main effect) with first-level stop signal (left) and nogo (right) effects. In the two rows below, empty maps illustrate the non-significant interactions between P3_RPE_ and OCI-R and NU scores (interaction effects; second and third rows, respectively). The last three rows show effects of the FRN_RPE_ (note that time points of topographies tied to P3_RPE_ and FRN_RPE_ differ)_._ Significant interactions between FRN_RPE_ and OCI-R (regression weights [b-values] from between-subjects analyses, red: positive, blue: negative, masked at *p* = 0.010), did not occur during meaningful EEG activity related to stopping (P3_stop_ emerged after 400 ms, see Fig. 2B) or withholding (P3_nogo_ emerged after 300 ms, see Fig 2A).

**Second Supplementary Hypothesis**

Based on our previous data (^4^), we pre-registered the hypothesis that moderation of the relationship between brain activity associated with feedback processing and motor inhibition by compulsivity and negative urgency should be specific for the P3b_MIFLAT_ (rather than P3a_MIFLAT_ or feedback-related negativity [FRN_MIFLAT_ and FRN_RPE_]) and the inhibition-related P3_stop_ and P3_nogo_ (rather than inhibition-related N2) in the SST and GNGT.

Methods

In the feedback-locked EEG, components other than the parietal P3b are frequently studied. The frontocentral feedback-related negativity (FRN) emerges around 250 ms after feedback onset with more negative amplitudes for outcomes worse than expected (^8^), and the P3a is a central, earlier aspect of the P3 complex thought to capture attentional orientation (^9^). We previously observed that the P3b_MIFLAT_, but not the FRN_MIFLAT_ or P3a_MIFLAT_, predicted P3_stop_ and P3_nogo_ in high binge-watching individuals (^4^). In order to test whether interactions of compulsivity and negative urgency were similarly specific to the P3b_MIFLAT_, we aimed to examine the role of EEG activity related to the FRN_MIFLAT_ and P3a_MIFLAT_ and their respective interactions with compulsivity and negative urgency. As shown in Fig. 2C, in the MIFLAT, we observed a frontocentral feedback-related negativity (FRN_MIFLAT_; FC4 peak at 264 ms, *t*(204) = -11.29, *p* < 10^-27^, critical *p* = .016, 98.36% CI [-0.34, -0.02], *g* = -0.79) and a central P3a (P3a_MIFLAT_; Cz peak at 408 ms, *t*(204) = 22.52, *p* < 10^-56^, critical *p* = .016, 98.36% CI [3.39, 4.21], *g* = 1.57) sensitive to valence. Therefore, as additional feedback effects in second-level analyses using Equations (5-7), we used the mean of first-level b-values of the feedback valence regressor in the MIFLAT, corresponding to the FRN_MIFLAT_ (FC4, 260-270 ms) and P3a_MIFLAT_ (Cz, 400-420 ms). As can be seen in Figure S1A, we also observed a frontocentral reward prediction error feedback-related negativity (FRN_RPE_; FCz peak at 262 ms, *t*(204) = 16.68, *p* < 10^-39^, critical *p* = .014, 99% CI [1.59 2.14], *g* = 1.16). Thus, in analogy, we examined EEG activity associated with the FRN_RPE_ from the two-step task (FCz, 256-266 ms). Reward prediction error did not have a distinct reflection in the P3a in the two-step task (see Fig. S1A).

Based on Equation (5), to predict the stop signal effect, we regressed b-values of the SST trial type regressor at each electrode and time point on (a) mean first-level b-values representing the FRN_MIFLAT_ and on their interaction with compulsivity, (b) mean first-level b-values representing the P3a_MIFLAT_ and on their interaction with compulsivity, and (c) mean first-level b-values representing the FRN_RPE_ and on their interaction with compulsivity (masked at *p* = .010). We applied the same procedure to predict the nogo effect (GNGT trial type regressor). We then repeated the same second-level procedure for negative urgency, based on Equation (6).

Results

Effects of the FRN_MIFLAT_ and its Interactions with Compulsivity on Inhibition

*Stop Signal Task.* A significant occipital relationship between the FRN_MIFLAT_ regressor and the stop signal effect in the P3_stop_ emerged (O2 peak at 476 ms, β = -0.63, 99% CI [-0.88 -0.37], *t*(204) = -4.34, *p* = 4.56 x 10^-5^; Fig. S1A), but the interaction with OCI-R was not significant (IO1 peak at 416 ms, β = 0.05, 99% CI [0.03 0.07], *t*(204) = 5.06, *p* = .047).

*Go/Nogo Task.* A significant relationship between the FRN_MIFLAT_ regressor and the nogo effect in the P3_nogo_ emerged (Cz peak at 342 ms, β = -0.57, 99% CI [-1.08 -0.06], *t*(204) = -3.83, *p* = .004; Fig. S1B), but no significant interaction with OCI-R was observed (FP1 peak at 294 ms, β = 0.03, 99% CI [0.02 0.04], *t*(204) = 2.65, *p* = .038).


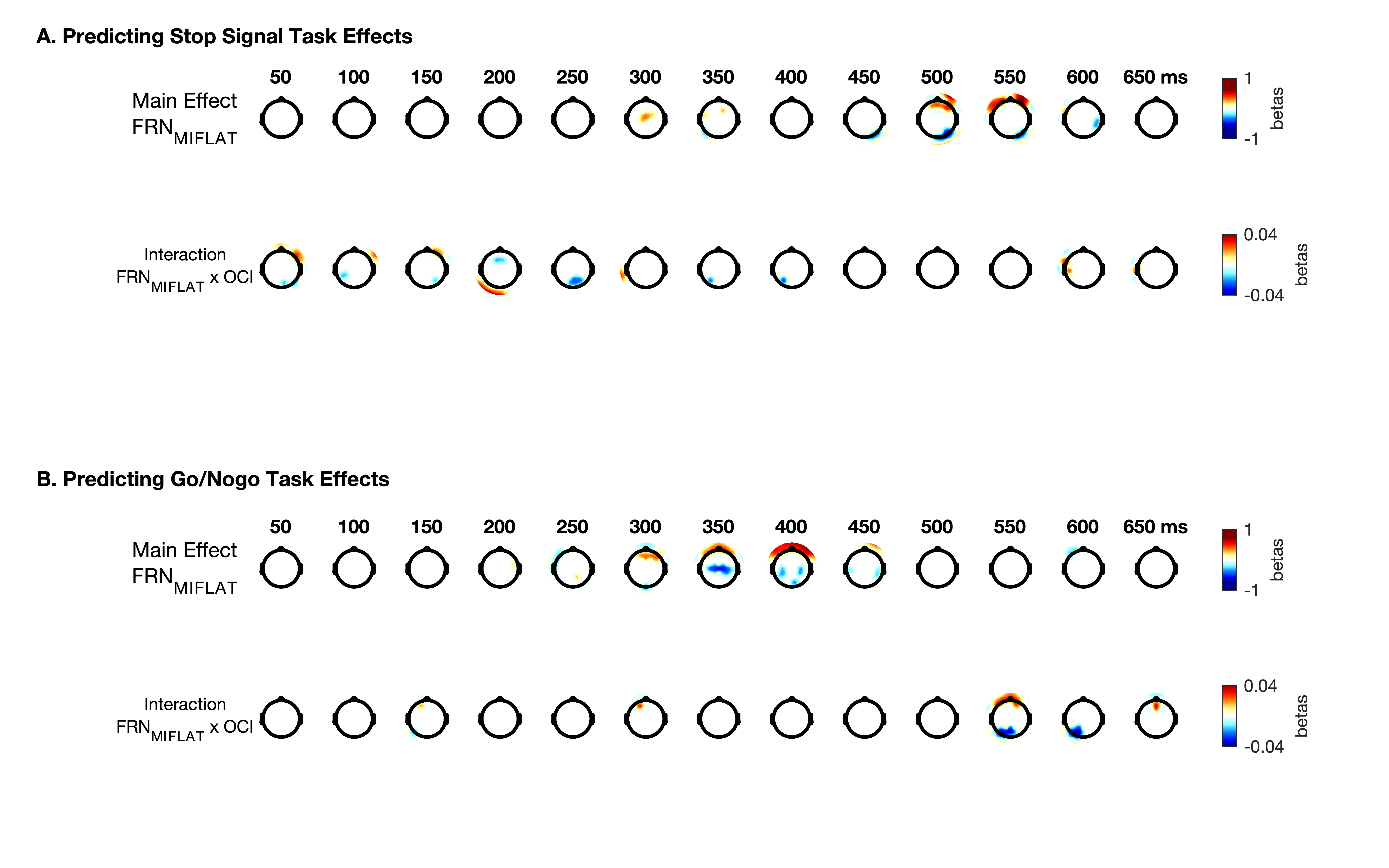


Figure S2. The (first-level) feedback-related negativity (FRN_MIFLAT_) predicts (first-level) stop signal effects in the stop signal task (A) and (first-level) nogo effects in the go/nogo task (B), but its interaction with compulsivity (OCI; Obsessive-Compulsive Inventory) did not modulate these main effects. In each figure section, topographical maps present significant associations of the FRN_MIFLAT_ (main effect; first row) as well as the interaction between FRN_MIFLAT_ and OCI scores (interaction effect; second row) with first-level stop signal (A) and nogo (B) effects. All topographical maps display regression weights (betas) from between-subjects analyses, red: positive, blue: negative, masked at *p* = 0.010.

Effects of the FRN_MIFLAT_ and its Interactions with Negative Urgency on Inhibition

*Stop Signal Task.* A significant occipital association between the FRN_MIFLAT_ regressor and the stop signal effect in the P3_stop_ was found (O2 peak at 476 ms, β = -0.65, 99% CI [-0.91 -0.38], *t*(204) = -4.33, *p* = 4.47 x 10^-5^; Fig. S2A). Additionally, the interaction term with negative urgency modulated the main effect (Cz peak at 530 ms, β = 0.11, 99% CI [0.04 0.19], *t*(204) = 4.59, *p* = 8.48 x 10^-5^). According to JN analyses, the central relationship between FRN_MIFLAT_ and P3_stop_ was significantly negative for negative urgency scores < 20.7 and turned significantly positive for urgency scores > 31.6.

*Go/Nogo Task* A significant relationship between the FRN_MIFLAT_ regressor and the nogo effect in the P3_nogo_ emerged (Cz peak at 344 ms, β = -0.63, 99% CI [-1.14 -0.12], *t*(204) = -4.10, *p* = .002; Fig. S2B), as well as a significant interaction with negative urgency (Cz peak at 430 ms, β = 0.08, 99% CI [0.02 0.14], *t*(204) = 3.77, *p* = 4.49 x 10^-4^). JN analyses indicated that the central relationship between FRN_MIFLAT_ and P3_nogo_ was significantly negative for negative urgency scores < 23.7 and turned significantly positive for urgency scores > 37.8


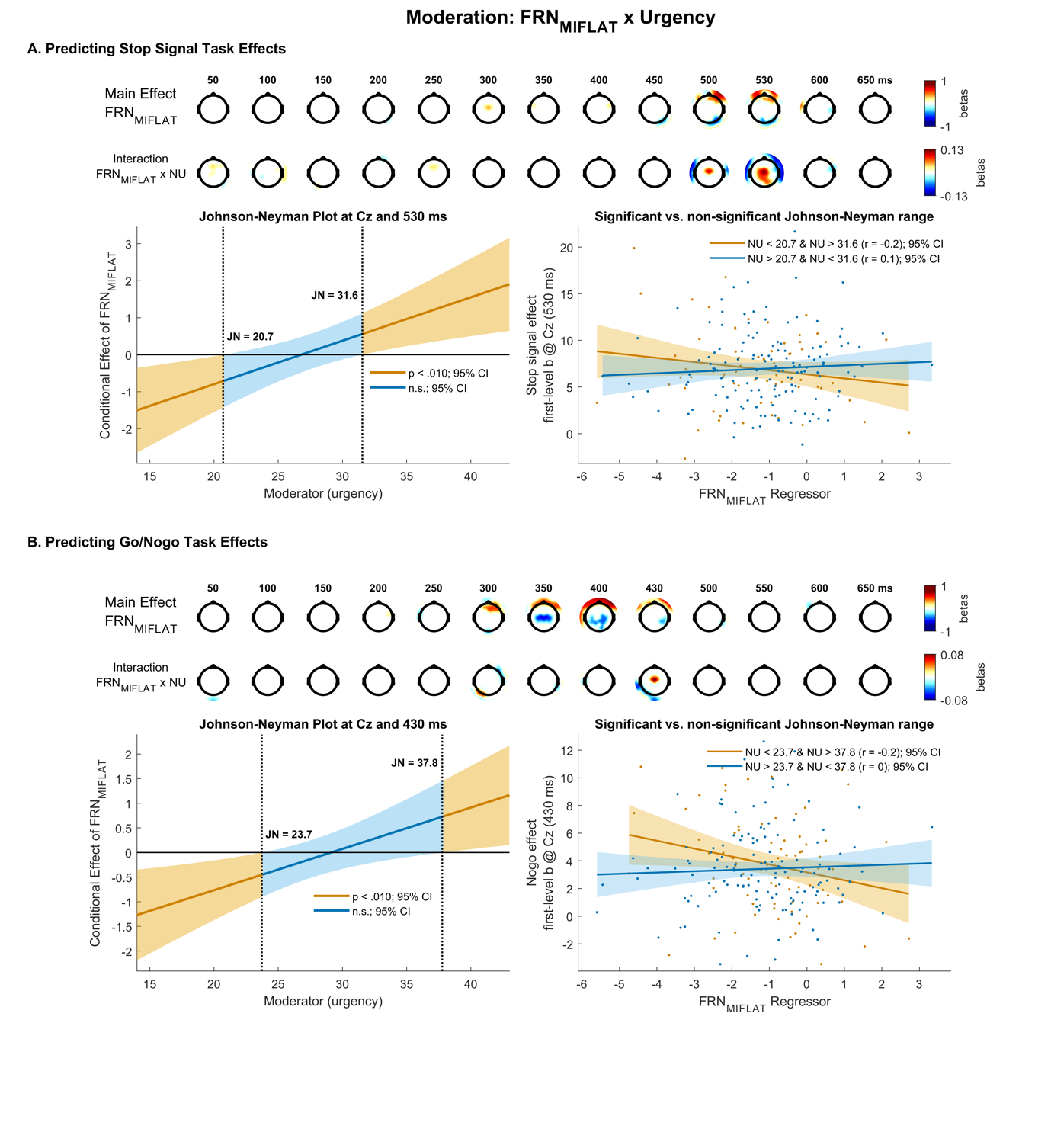


Figure S3. The (first-level) feedback-related negativity (FRN_MIFLAT_) and its interaction with negative urgency (NU) predict (first-level) stop signal effects in the stop signal task (A) and (first-level) nogo effects in the go/nogo task (B). In each figure section, the two top rows present topographical maps of significant associations of the FRN_MIFLAT_ (main effect; first row) as well as the interaction between FRN_MIFLAT_ and NU scores (interaction effect; second row) with first-level stop signal (A) and nogo (B) effects. The lower left panels show Johnson-Neyman (JN) plots depicting simple slopes of the main effect as a function of score on the moderator, with vertical lines indicating JN points at which the main effect switches from significant (orange) to non-significant (blue). The lower right panels show scatterplots, contrasting NU groups scoring below and above vs. between the JN points, of the relationship between the FRN_MIFLAT_ regressor as entered into the model (Equation 6) and first-level b-values for the stop signal (A) and nogo (B) effect at a central electrode and time point where the interaction effect of the FRN_MIFLAT_ with NU was maximal. All topographical maps display regression weights (betas) from between-subjects analyses, red: positive, blue: negative, masked at *p* = 0.010.

Effects of the FRN_RPE_ and its Interactions with Compulsivity and Urgency on Inhibition

*Stop Signal Task.* A significant interaction of the FRN_RPE_ regressor with OCI-R was observed before the P3_stop_ developed (PO2 peak at 402 ms, β = 0.04, 99% CI [0.01 0.07], *t*(204) = 4.34, *p* = 3.38 x 10^-5^; main effect: FPz peak at 438 ms, β = -0.42, 99% CI [-0.53 -0.30], *t*(204) = -3.54, *p* = .004; Fig. S1B). A significant interaction of the FRN_RPE_ regressor with negative urgency was observed after meaningful stopping-related EEG activity (Oz peak at 724 ms, β = 0.08, 99% CI [0.03 0.13], *t*(204) = 4.17, *p* = 4.58 x 10^-5^; main effect: F1 peak at 526 ms, β = -0.40, 99% CI [-0.69 -0.12], *t*(204) = -3.62, *p* = 3.80 x 10^-4^) and was therefore not interpreted.

*Go / Nogo Task.* A significant interaction of the FRN_RPE_ regressor with OCI-R began to develop between the N2 and the P3_nogo_ and peaked during early ramp-up of the P3_nogo_ (Pz peak at 358 ms, β = 0.04, 99% CI [0.01 0.07], *t*(204) = 4.00, *p* = 3.15 x 10^-4^; main effect: Cz peak at 220 ms, β = -0.37, 99% CI [-0.60 -0.14], *t*(204) = -4.49, *p* = 5.52 x 10^-5^; Fig. S1B) and was not interpreted. A significant interaction of the FRN_RPE_ regressor with negative urgency was observed (PO2 peak at 456 ms, β = 0.06, 99% CI [0.02 0.11], *t*(204) = 4.01, *p* = 7.19 x 10^-4^), but temporospatially distinct from the main effect (FCz peak at 400 ms, β = -0.37, 99% CI [-0.69 -0.04], *t*(204) = -4.58, *p* = .004) and was therefore not interpreted.

Effects of the P3a_MIFLAT_ and its Interactions with Compulsivity on Inhibition

*Stop Signal Task*. A significant relationship between the P3a_MIFLAT_ regressor and the stop signal effect in the P3_stop_ emerged (Cz peak at 466 ms, β = 0.71, 99% CI [0.43 0.99], *t*(204) = 4.26, *p* = 6.04 x 10^-10^; Fig. S3A), and a significant interaction with OCI-R was observed (CP2 peak at 462 ms, β = -0.026, 99% CI [-0.03 -0.02], *t*(204) = -3.53, *p* = .006), with JN analyses indicating that the relationship between the P3a_MIFLAT_ regressor and the stop signal effect was significantly positive for mild to moderate OCI-R scores < 22.8.

*Go/Nogo Task.* A significant relationship between the P3a_MIFLAT_ regressor and the nogo effect in the P3_nogo_ emerged (Cz peak at 316 ms, β = 0.48, 99% CI [0.41 0.54], *t*(204) = 4.34, *p* = 4.59 x 10^-5^; Fig. S3B), but no significant interaction with OCI-R was observed (Cz peak at 338 ms, β = -0.02, 99% CI [-0.03 -0.01], *t*(204) = -2.99, *p* = .054).


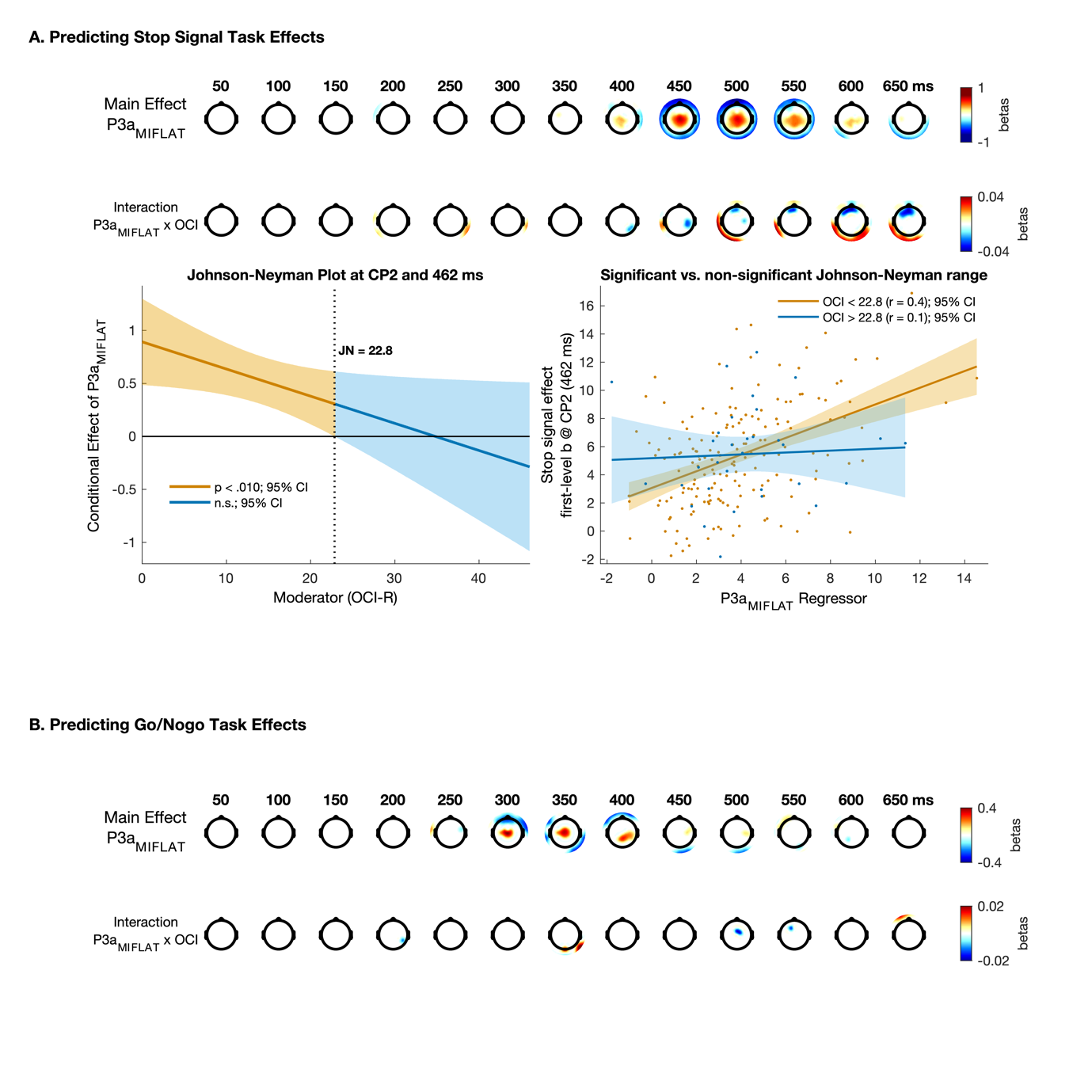


Figure S3. The (first-level) feedback-related P3a (P3a_MIFLAT_) and its interaction with compulsivity (OCI; Obsessive-Compulsive Inventory) predicts (first-level) stop signal effects in the stop signal task (A) but not (first-level) nogo effects in the go/nogo task (B). In each figure section, topographical maps present significant associations of the P3a_MIFLAT_ (main effect; first row) as well as the interaction between P3a_MIFLAT_ and OCI scores (interaction effect; second row) with first-level stop signal (A) and nogo (B) effects. The lower left panel in (A) shows a Johnson-Neyman (JN) plot depicting the simple slope of the main effect as a function of score on the moderator, with the vertical line indicating the JN point at which the main effect switches from significant (orange) to non-significant (blue). The lower right panel in (A) shows a scatterplot, contrasting OCI groups scoring below and above the JN point, of the relationship between the P3a_MIFLAT_ regressor as entered into the model (Equation 6) and first-level b-values for the stop signal effect at a centroparietal electrode and time point where the interaction effect of the Pab_MIFLAT_ with compulsivity was maximal. All topographical maps display regression weights (betas) from between-subjects analyses, red: positive, blue: negative, masked at *p* = 0.010.

Effects of the P3a_MIFLAT_ and its Interactions with Negative Urgency on Inhibition

*Stop Signal Task*. The relationship between the P3a_MIFLAT_ regressor and the stop signal effect in the P3_stop_ (Cz peak at 466 ms, β = 0.64, 99% CI [0.36 0.93], *t*(204) = 6.42, *p* = 2.28 x 10^-8^; Fig. S4A) was not qualified by an interaction with negative urgency (IO1 peak at 448 ms, β = -0.03, 99% CI [-0.10 -0.03], *t*(204) = -3.84, *p* = .172).

*Go/Nogo Task.* The relationship between the P3a_MIFLAT_ regressor and the nogo effect in the P3_nogo_ (Cz peak at 316 ms, β = 0.47, 99% CI [0.18 0.76], *t*(204) = 4.49, *p* = 5.24 x 10^-5^; Fig. S4B) was not qualified by an interaction with negative urgency (FCz peak at 262 ms, β = -0.04, 99% CI [-0.08 0.0008], *t*(204) = -3.25, *p* = .012).


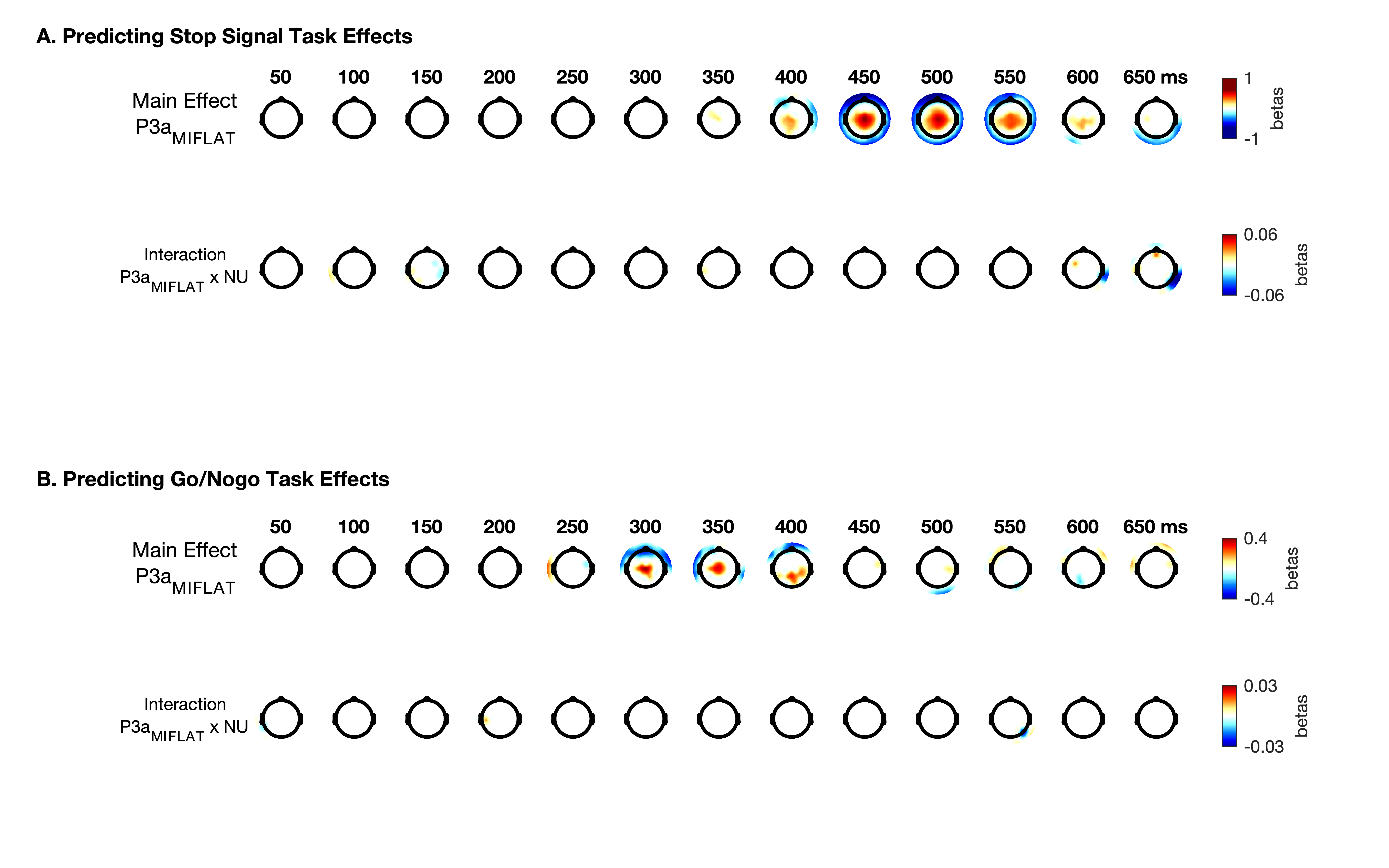


Figure S4. The (first-level) feedback-related P3a (P3a_MIFLAT_) predicts (first-level) stop signal effects in the stop signal task (A) and (first-level) nogo effects in the go/nogo task (B), but its interaction with negative urgency (NU) did not modulate these main effects. In each figure section, topographical maps present significant associations of the P3a_MIFLAT_ (main effect; first row) as well as the interaction between P3a_MIFLAT_ and NU scores (interaction effect; second row) with first-level stop signal (A) and nogo (B) effects. All topographical maps display regression weights (betas) from between-subjects analyses, red: positive, blue: negative, masked at *p* = 0.010.

In sum, consistent with pre-registered hypotheses and our prior study (^4^), moderating effects were specific to the P3_stop_ as well as P3_nogo_, which are assumed to reflect the actual implementation of inhibition (^10,11^). Moderation was also most robust for the P3b_MIFLAT_ regressor. The P3b is assumed to capture the motivational salience as well as late computations of expectancy violations (^12,13^) and is sensitive to subsequent behavioral adaptations (^14^), which is suggestive of the behavioral significance of our findings. However, contrasting our supplementary Hypothesis 2, FRN_MIFLAT_ and its interaction with negative urgency (but not OCI-R) replicated the pattern of the P3b_MIFLAT_ that the relationship between systems was present in those with low, but not high expressions of negative urgency for both motor inhibition tasks. The FRN is considered an index of early computations regarding expectancy violations. The finding that particularly early feedback-related signals show an effect in negative urgency is consistent with the rash, premature behaviors captured by this construct. Also, compulsivity moderated the relationship between the P3a_MIFLAT_ and the P3_stop_. This indicates a minor role of attentional orientation, which is captured by the P3a (^9^), in systems imbalance.

**Exploratory Analyses**

Further, in order to contextualize our results within literature concerned with main effects of high vs. low impulsive-compulsive phenotypes within isolated psychological domains (^15-18^), we conducted regression analyses using compulsivity and negative urgency scores (“questionnaire” in Equation 8) as predictors for EEG activity associated with task effects in the SST, GNGT, MIFLAT, and two-step task (“First-level b” in Equation 8), according to the following formula:

First-level b = β_0_ + β_1_ x *questionnaire* + *Error* (8)

In order to control whether the currently observed moderation effects were specific to compulsivity and negative urgency, we considered the other impulsivity facets contained in the UPPS Scale, i.e. lack of premeditation (Cronbach’s α = 0.74, *M*±*SD* = 22.3±4.5; theoretical range: 11 - 44, actual range: 12 - 38), lack of perseverance (Cronbach’s α = 0.81, *M*±*SD* = 19.4±4.4, theoretical range: 10 - 40, actual range: 10 – 31), and sensation seeking (Cronbach’s α = 0.84, *M*±*SD* = 32.8±7.0, theoretical range: 12 - 48, actual range: 14 - 46), as potential moderators of the relationship between the P3b_MIFLAT_ and the P3_stop_ as well as the P3_nogo_. For this purpose, we exchanged the negative urgency scores in the interaction term in Equation (6) for each of the other UPPS subscales and reran the second-level regressions. This is a pre-registered exploratory analysis.

Lastly, the OCI-R captures OC symptoms on the following subscales (theoretical range of each: 0 - 12): checking (Cronbach’s α = 0.85, *M*±*SD* = 2.3±2.5; range: 0 - 12), hoarding (Cronbach’s α = 0.79, *M*±*SD* = 2.3±2.1; range: 0 - 10), neutralizing (Cronbach’s α = 0.61, *M*±*SD* = 1.0±1.6; range: 0 - 9), ordering (Cronbach’s α = 0.90, *M*±*SD* = 3.2±2.7; range: 0 - 12), obsessing (Cronbach’s α = 0.82, *M*±*SD* = 2.3±2.3; range: 0 - 9), and washing (Cronbach’s α = 0.77, *M*±*SD* = 1.5±2.0; range: 0 - 10). In order to explore which subscales yielded moderation effects, we reran second-level regressions based on Equation (5), replacing OCI-R sum scores with scores from each subscale. These analyses are exploratory and not pre-registered, and some subscales showed insufficient distributions.

Results

Main effects of compulsivity and negative urgency on stopping, withholding, and feedback processing

Compulsivity was associated with higher loss vs. gain activity tied to the P3a_MIFLAT_ (FC1 peak at 356 ms, β = 0.06, *t*(204) = 2.72, *p* = .007), but did not meaningfully modulate brain activity tied to the reward prediction error signal in the two-step task (T2 peak at 324 ms, β = 0.03, *t*(204) = 2.91, *p* = .004; note that we already published this analysis in the full two-step sample in ^7^), stopping (C4 peak at 258 ms, β = -0.02, *t*(204) = -2.73, *p* = .007; note that this was already published in the full SST sample in ^19^) or withholding (FC3 peak at 788 ms, β = -0.04, *t*(204) = -3.79, *p* = 2.01 x 10^-4^).

In contrast, negative urgency did not modulate brain activity associated with feedback processing in the MIFLAT (F5 peak at 136 ms, β = 0.04, *t*(204) = 2.76, *p* = .006), but was related to lower reward prediction error signal associated with the P3_RPE_ in the two-step task (PO4 peak at 388 ms, β = -0.08, *t*(204) = -4.90, *p* = 1.99 x 10^-6^) as well as lower successful stop vs. go (F1 peak at 518 ms, β = -0.09, *t*(204) = -3.56, *p* = 4.68 x 10^-4^; note that we already published this effect with the full SST sample in ^19^) and nogo vs. go (FCz peak at 400 ms, β = -0.13, *t*(204) = -3.63, *p* = 3.59 x 10^-4^) activity tied to the P3_stop_ and P3_nogo_ in the SST and GNGT, respectively.

In sum, when considering associations of compulsivity and negative urgency separately within each task, they exhibited mutually exclusive relationships with task-related brain activity. This is in contrast to the substantial overlap between compulsivity and negative urgency regarding the moderation of brain-brain relationships of the P3b_MIFLAT_ with nogo and stop signal-related brain activity.

Moderating Effects of Other Impulsivity Facets from the UPPS Scale

In brief, we observed that none of the other UPPS facets were significant moderators in the SST (Table S2) or GNGT (Table S2).

| **Table S1.**  *Overview of Peak Effects in Models Incorporating Other UPPS Subscales as Moderators in Predicting Stop Signal Task EEG Activity.* | | | | | | |
| --- | --- | --- | --- | --- | --- | --- |
| Effect | Electrode | Time (ms) | β | 99% CI | t | p |
| Model Lack of Perseverance |  |  |  |  |  |  |
| P3b_MIFLAT_ | Pz | 524 | 0.52 | [0.30 0.73] | 7.20 | 2.32 x 10^-9^ |
| P3b_MIFLAT_ x Perseverance | IO2 | 554 | -0.05 | [-0.11 0.02] | -4.89 | .060 |
| Model Lack of Premeditation |  |  |  |  |  |  |
| P3b_MIFLAT_ | Pz | 606 | 0.49 | [0.30 0.68] | 6.63 | 2.94 x 10^-10^ |
| P3b_MIFLAT_ x Premeditation | Cz | 538 | -0.06 | [-0.11 0.002] | -3.53 | .013 |
| Model Sensation Seeking |  |  |  |  |  |  |
| P3b_MIFLAT_ | Pz | 602 | 0.51 | [0.32 0.69] | 7.13 | 1.78 x 10^-11^ |
| P3b_MIFLAT_ x Sensation Seeking | AF4 | 730 | -0.04 | [-0.06 -0.01] | -4.52 | 5.83 x 10^-5^ |

| **Table S2.**  *Overview of Peak Effects in Models Incorporating Other UPPS Subscales as Moderators in Predicting Go/Nogo Task EEG Activity.* | | | | | | |
| --- | --- | --- | --- | --- | --- | --- |
| Effect | Electrode | Time (ms) | β | 99% CI | t | p |
| Model Lack of Perseverance |  |  |  |  |  |  |
| P3b_MIFLAT_ | Cz | 316 | 0.43 | [0.11 0.74] | 3.95 | 5.22 x 10^-4^ |
| P3b_MIFLAT_ x Perseverance | PO2 | 288 | -0.04 | [-0.09 0.002] | -3.27 | .014 |
| Model Lack of Premeditation |  |  |  |  |  |  |
| P3b_MIFLAT_ | Cz | 324 | 0.49 | [0.17 0.81] | 3.93 | 1.21 x 10^-4^ |
| P3b_MIFLAT_ x Premeditation | LO1 | 390 | -0.04 | [-0.09 0.002] | -3.99 | .015 |
| Model Sensation Seeking |  |  |  |  |  |  |
| P3b_MIFLAT_ | Cz | 312 | 0.45 | [0.14 0.75] | 3.79 | 2.12 x 10^-4^ |
| P3b_MIFLAT_ x Sensation Seeking | PO10 | 182 | 0.03 | [-0.05 -0.009] | -3.98 | 1.85 x 10^-4^ |

Moderating Effects of OCI-R Symptom Subscales

*Stop Signal Task*. In brief, we observed that all OCI-R subscales except checking were significant moderators (see Table S3).

| **Table S3.**  *Overview of Peak Effects in Models Incorporating OCI-R Subscales as Moderators in Predicting Stop Signal Task EEG Activity.* | | | | | | | |
| --- | --- | --- | --- | --- | --- | --- | --- |
| Effect | Electrode | Time (ms) | β | 99% CI | t (204) | p | JN points^a^ |
| Model Checking |  |  |  |  |  |  |  |
| P3b_MIFLAT_ | Pz | 594 | 0.52 | [0.32 0.71] | 6.85 | 1.02 x 10^-10^ | — |
| P3b_MIFLAT_ x Checking | P10 | 750 | -0.07 | [-0.13 0.003] | -3.38 | .015 | — |
| Model Washing |  |  |  |  |  |  |  |
| P3b_MIFLAT_ | Pz | 602 | 0.53 | [0.34 0.71] | 7.45 | 2.75 x 10^-12^ | — |
| P3b_MIFLAT_ x Washing | PO1 | 530 | -0.18 | [-0.27 -0.09] | -5.05 | 1.07 x 10^-6^ | 2.7, 6.9 |
| Model Hoarding |  |  |  |  |  |  |  |
| P3b_MIFLAT_ | Pz | 596 | 0.53 | [0.34 0.72] | 7.18 | 1.13 x 10^-11^ | — |
| P3b_MIFLAT_ x Hoarding | Pz | 534 | -0.15 | [-0.23 -0.06] | -5.55 | 2.70 x 10^-5^ | 4.0 |
| Model Obsessing |  |  |  |  |  |  |  |
| P3b_MIFLAT_ | Pz | 594 | 0.55 | [0.36 0.73] | 7.61 | 1.17 x 10^-12^ | — |
| P3b_MIFLAT_ x Obsessing | CPz | 492 | -0.14 | [-0.25 -0.03] | -3.83 | 7.45 x 10^-4^ | 3.90 |
| Model Ordering |  |  |  |  |  |  |  |
| P3b_MIFLAT_ | Pz | 596 | 0.52 | [0.33 0.71] | 7.15 | 1.83 x 10^-11^ | — |
| P3b_MIFLAT_ x Ordering | Cz | 566 | -0.08 | [-0.16 -0.002] | -4.03 | .009 | 4.8 |
| Model Neutralizing |  |  |  |  |  |  |  |
| P3b_MIFLAT_ | CPz | 526 | 0.52 | [0.30 0.74] | 7.14 | 9.47 x 10^-9^ | — |
| P3b_MIFLAT_ x Neutralizing | Cz | 550 | -0.22 | [-0.34 -0.10] | -6.46 | 8.74 x 10^-6^ | 1.6, 5.2 |
| ^a^ Johnson-Neyman points: Simple slope is significantly positive below the first entry and significantly negative above second entry, if applicable. | | | | | | | |

*Go / Nogo Task*. We observed that all OCI-R subscales except ordering and obsessing were significant moderators (see Table S4).

| **Table S4.**  *Overview of Peak Effects in Models Incorporating OCI-R Subscales as Moderators in Predicting Go/Nogo Task EEG Activity.* | | | | | | | |
| --- | --- | --- | --- | --- | --- | --- | --- |
| Effect | Electrode | Time (ms) | β | 99% CI | t (204) | p | JN points^a^ |
| Model Checking |  |  |  |  |  |  |  |
| P3b_MIFLAT_ | Cz | 324 | 0.46 | [0.13 0.79] | 3.86 | 4.01 x 10^-4^ | — |
| P3b_MIFLAT_ x Checking | CP4 | 342 | -0.09 | [-0.14 -0.04] | -4.63 | 2.64 x 10^-5^ | 4.6 |
| Model Washing |  |  |  |  |  |  |  |
| P3b_MIFLAT_ | Cz | 316 | 0.46 | [0.15 0.77] | 3.88 | 1.67 x 10^-4^ | — |
| P3b_MIFLAT_ x Washing | Cz | 336 | -0.15 | [-0.28 -0.02] | -4.62 | .003 | 2.3 |
| Model Hoarding |  |  |  |  |  |  |  |
| P3b_MIFLAT_ | Cz | 312 | 0.49 | [0.17 0.80] | 3.98 | 9.68 x 10^-5^ | — |
| P3b_MIFLAT_ x Hoarding | FC2 | 336 | -0.13 | [-0.25 -0.02] | -4.57 | .003 | 2.7 |
| Model Obsessing |  |  |  |  |  |  |  |
| P3b_MIFLAT_ | Cz | 322 | 0.43 | [0.10 0.75] | 3.78 | 7.62 x 10^-4^ | — |
| P3b_MIFLAT_ x Obsessing | P10 | 200 | -0.08 | [-0.15 -0.003] | -3.71 | .008 | — |
| Model Ordering |  |  |  |  |  |  |  |
| P3b_MIFLAT_ | Cz | 322 | 0.45 | [0.13 0.77] | 3.73 | 4.18 x 10^-4^ | — |
| P3b_MIFLAT_ x Ordering | CP2 | 334 | -0.07 | [-0.16 0.01] | -5.64 | .027 | — |
| Model Neutralizing |  |  |  |  |  |  |  |
| P3b_MIFLAT_ | Cz | 312 | 0.43 | [0.12 0.73] | 3.73 | 3.94 x 10^-4^ | — |
| P3b_MIFLAT_ x Neutralizing | Oz | 294 | -0.20 | [-0.29 -0.10] | -5.63 | 3.50 x 10^-7^ | 0.6 2.8 |
| ^a^ Johnson-Neyman points: Simple slope is significantly positive below the first entry and significantly negative above second entry, if applicable. | | | | | | | |

**Internal Consistency of EEG Data**

Table S5 presents internal consistencies of various event-related potentials, using Spearman-Brown corrected split-half reliabilities (odd-even method).

| **Table S5**.  *Internal consistencies of Event-related Potentials.* | | | |
| --- | --- | --- | --- |
| Task | P3 | P3a | FRN |
| Go / Nogo^a^ |  |  |  |
| Go | 0.97 | - | - |
| Nogo | 0.94 | - | - |
| Stop Signal^b^ |  |  |  |
| Go | 0.91 | - | - |
| Stop | 0.80 | - | - |
| MIFLAT^c^ |  |  |  |
| Gain | 0.94 | 0.97 | 0.96 |
| Loss | 0.88 | 0.94 | 0.90 |
| Two-step^d^ |  |  |  |
| Gain | 0.96 | - | - |
| Loss | 0.90 | - | - |
| RPE^e^ | 0.98 | - | 0.98 |
| *Note*. MIFLAT = monetary incentive flanker task, FRN = feedback-related negativity, RPE = reward prediction error.  ^a^ FC1, 360 – 380 ms for P3_nogo_.  ^b^ P2, 560 – 580 ms for P3_stop_.  ^c^ Pz, 500 – 520 ms for P3b_MIFLAT_; Cz, 400-420 ms for P3a_MIFLAT;_ FC4, 260-270 ms for FRN_MIFLAT_.  ^d^ CP1, 440 – 460 for P3_two-step_.  ^e^ FCz, 256 – 266 ms for FRN_RPE_; CP5, 380 – 390 for P3_RPE_. | | | |

References

1. Verbruggen, F, Aron, AR, Band, GPH, Beste, C, Bissett, PG, Brockett, AT *et al*. A consensus guide to capturing the ability to inhibit actions and impulsive behaviors in the stop-signal task. *Elife* <https://doi.org/10.7554/eLife.46323> (2019).

2. Aron, AR & Poldrack, RA. Cortical and subcortical contributions to stop signal response inhibition: Role of the subthalamic nucleus. *Journal of Neuroscience* <https://doi.org/10.1523/Jneurosci.4682-05.2006> (2006).

3. Endrass, T, Schuermann, B, Kaufmann, C, Spielberg, R, Kniesche, R & Kathmann. Performance monitoring and error significance in patients with obsessive-compulsive disorder. *Biol Psychol* <https://doi.org/10.1016/j.biopsycho.2010.02.002> (2010).

4. Dieterich, R, Wüllhorst, V, Berghäuser, J, Overmeyer, R & Endrass, T. Electrocortical correlates of impaired motor inhibition and outcome processing are related in high binge-watching. *Psychophysiology* <https://doi.org/10.1111/psyp.13814> (2021).

5. Weafer, J, Crane, NA, Gorka, SM, Phan, KL & de Wit, H. Neural correlates of inhibition and reward are negatively associated. *Neuroimage* <https://doi.org/10.1016/j.neuroimage.2019.04.021> (2019).

6. Gillan, CM, Kosinski, M, Whelan, R, Phelps, EA & Daw, ND. Characterizing a psychiatric symptom dimension related to deficits in goal-directed control. *Elife* <https://doi.org/10.7554/eLife.11305> (2016).

7. Dück, K, Wüllhorst, R, Overmeyer, R & Endrass, T. On the effects of impulsivity and compulsivity on neural correlates of model-based performance. *Scientific Reports* <https://doi.org/10.1038/s41598-024-71692-w> (2024).

8. Sambrook, TD & Goslin, J. A Neural Reward Prediction Error Revealed by a Meta-Analysis of ERPs Using Great Grand Averages. *Psychological Bulletin* <https://doi.org/10.1037/bul0000006> (2015).

9. Polich, J. Updating p300: An integrative theory of P3a and P3b. *Clinical Neurophysiology* <https://doi.org/10.1016/j.clinph.2007.04.019> (2007).

10. Enriquez-Geppert, S, Konrad, C, Pantev, C & Huster, RJ. Conflict and inhibition differentially affect the N200/P300 complex in a combined go/nogo and stop-signal task. *Neuroimage* <https://doi.org/10.1016/j.neuroimage.2010.02.043> (2010).

11. Waller, DA, Hazeltine, E & Wessel, JR. Common neural processes during action-stopping and infrequent stimulus detection: The frontocentral P3 as an index of generic motor inhibition. *International Journal of Psychophysiology* <https://doi.org/10.1016/j.ijpsycho.2019.01.004> (2021).

12. Mars, RB, Debener, S, Gladwin, TE, Harrison, LM, Haggard, P, Rothwell, JC *et al*. Trial-by-Trial Fluctuations in the Event-Related Electroencephalogram Reflect Dynamic Changes in the Degree of Surprise. *Journal of Neuroscience* <https://doi.org/10.1523/Jneurosci.2925-08.2008> (2008).

13. Yeung, N & Sanfey, AG. Independent coding of reward magnitude and valence in the human brain. *Journal of Neuroscience* <https://doi.org/10.1523/Jneurosci.4537-03.2004> (2004).

14. Fischer, AG & Ullsperger, M. Real and Fictive Outcomes Are Processed Differently but Converge on a Common Adaptive Mechanism. *Neuron* <https://doi.org/10.1016/j.neuron.2013.07.006> (2013).

15. Le, TM, Potvin, S, Zhornitsky, S & Li, CSR. Distinct patterns of prefrontal cortical disengagement during inhibitory control in addiction: A meta-analysis based on population characteristics. *Neuroscience and Biobehavioral Reviews* <https://doi.org/10.1016/j.neubiorev.2021.04.028> (2021).

16. Luijten, M, Schellekens, AF, Kühn, S, Machielse, MWJ & Sescousse, G. Disruption of Reward Processing in Addiction An Image-Based Meta-analysis of Functional Magnetic Resonance Imaging Studies. *Jama Psychiatry* <https://doi.org/10.1001/jamapsychiatry.2016.3084> (2017).

17. Rasgon, A, Lee, WH, Leibu, E, Laird, A, Glahn, D, Goodman, W *et al*. Neural correlates of affective and non-affective cognition in obsessive compulsive disorder: A meta-analysis of functional imaging studies. *European Psychiatry* <https://doi.org/10.1016/j.eurpsy.2017.08.001> (2017).

18. Thorsen, AL, Hagland, P, Radua, J, Mataix-Cols, D, Kvale, G, Hansen, B *et al*. Emotional Processing in Obsessive-Compulsive Disorder: A Systematic Review and Meta-analysis of 25 Functional Neuroimaging Studies. *Biological Psychiatry-Cognitive Neuroscience and Neuroimaging* <https://doi.org/10.1016/j.bpsc.2018.01.009> (2018).

19. Wüllhorst, V, Wüllhorst, R, Overmeyer, R & Endrass, T. Comprehensive Analysis of Event-Related Potentials of Response Inhibition: The Role of Negative Urgency and Compulsivity. *Psychophysiology* <https://doi.org/10.1111/psyp.70000> (2025).
